# Evidence standards for multi-ancestry polygenic prediction

**DOI:** 10.1186/s13059-026-04187-8

**Published:** 2026-07-13

**Authors:** Blessing Oselu, Itunuoluwa Isewon, Jelili Oyelade, Conrad O. Iyegbe

**Affiliations:** 1 Department of Computer and Information Sciences, Covenant University, P.M.B 1023, Ota, Ogun State Nigeria; 2 Covenant Applied Informatics and Communication African Centre of Excellence (CApIC-ACE), Covenant University, P.M.B 1023, Ota, Ogun State Nigeria; 3 Covenant University Bioinformatics Research (CUBRe), Covenant University, P.M.B 1023, Ota, Ogun State Nigeria; 4 Department of Genetics and Genomic Sciences, Icahn School of Medicine at Mount Sinai Hospital, New York, USA

## Abstract

Polygenic scores (PGS) are prospective tools for health screening, prevention and trials, but most are trained in European genome-wide association studies and lose accuracy and calibration in non-European populations and those of mixed ancestral heritage. Multi-ancestry methods are proliferating, yet benchmarking standards lag. This review focuses on benchmark design for cross-population prediction. We show how ancestry assignment, linkage disequilibrium references, variant sets, tuning and trait architecture shape apparent performance. We propose a scorecard spanning discrimination, calibration, equity gaps and compute cost, plus stress tests in diverse cohorts and realistic simulations. Finally. we outline a future vision of living, auditable benchmarking frameworks.

**Supplementary Information:**

The online version contains supplementary material available at https://doi.org/10.1186/s13059-026-04187-8.

## Introduction

Polygenic scores (PGS) summarise the genetic contribution to disease risk or trait variation by combining the estimated effects of many genetic variants into a single predictor [1]. We use PGS as the broad term throughout this review, reserving polygenic risk score (PRS) for clinical-risk contexts and for software names that use the term. Genome-wide association studies (GWAS) provide the effect estimates used to construct PGS. For traits with GWAS sample sizes in the millions, predictive performance has begun to approach levels consistent with clinical utility in some cases [2]. However, discovery GWAS remain disproportionately European [3, 4], which reduces the ability to reproduce headline PGS performance in non-European populations. Genetic distance between discovery and target populations has a direct relationship with cross-ancestry PGS portability [5]. This helps explain why European-trained scores show their largest performance declines in African-ancestry cohorts, demonstrating relative drops of approximately 80% in studies of 17 [6] and 245 [5] UK Biobank traits.

Convergent mechanisms drive these performance declines. Differences in linkage disequilibrium (LD; correlation structure between nearby genetic variants) and allele frequency spectra mean that variants which tag causal loci well in one ancestry may be weak proxies in another, reducing the captured signal. These differences are rooted in human population history. Modern humans originated in Africa, where the deepest genetic diversity is retained. The out-of-Africa bottleneck reduced diversity in non-African populations and reshaped allele frequency spectra and LD patterns. As a result of this African populations show greater genetic diversity and shorter-range LD, while non-African populations carry subsets of this diversity, which have been shaped by subsequent drift, migration and founder events [4, 6, 7]. Within Africa, deep population structure, migration, admixture, drift and local adaptation have also generated substantial heterogeneity in allele frequencies, LD patterns and ancestry composition among populations. Thus, continental African ancestry is not a uniform genetic construct and PGS performance can differ sharply between even neighbouring African populations [7–9].

Non-genetic influences are also important. Healthcare access, phenotype definitions, ascertainment and environmental risk exposures often differ across settings, shifting both relative and absolute risk when scores are deployed outside their training context [10, 11]. Differences in ascertainment, covariate adjustment and evaluation design can likewise shape apparent performance, leading studies of the same PGS to reach divergent conclusions [12, 13]. Together, these sources of heterogeneity make rigorous benchmarking essential for distinguishing true methodological gains from artefacts of context and design.

A carefully designed benchmarking process provides the experimental conditions needed to determine which approaches sustain strong discrimination and calibration across populations and settings, to measure the size of equity gaps and to evaluate the extent to which proposed new methods address those gaps [14] and support reproducibility through standardised workflows and transparent reporting [15]. Benchmarking is therefore an essential part of the evidentiary backbone needed to deliver responsible and equitable polygenic prediction. Several valuable resources already exist; Pain et al. introduced a reference-standardised evaluation framework and an accompanying workflow, GenoPred, that reduces hidden analytic variation and makes comparisons more reproducible [16]. Meanwhile, comprehensive reviews have already catalogued single-source and general-purpose PGS software [17, 18]. This review is complementary to those efforts. Rather than providing a software catalogue, this review asks how multi-ancestry PGS claims should be tested when prediction must generalise across ancestry gradients, admixed genomes, heterogeneous phenotypes and clinical contexts.

*Who is in need of multi-ancestry PGS benchmarking guidance?* While formal benchmarking is often conducted by method developers and specialist groups, the resulting benchmarks have application to a wide variety of stakeholders. For *the developer*, it is important for defining incremental value; *cohort analysts* use performance benchmarks to decide what tools to analyse with, what reference panel to choose and how to tune algorithms; *biobanks, repositories and clinical implementation* studies increasingly depend on validation and performance evidence when deciding which polygenic scores are suitable for dissemination, evaluation or downstream use [19]; *clinical researchers* use them to interpret calibration and subgroup validity; finally, *reviewers, editors, consortia* and *funders* will often consult benchmarks when judging the credibility of results claimed. Multi-ancestry benchmarking is therefore part of the wider trust infrastructure required for responsible polygenic prediction-making. This review is organised around five practical questions: First, what is being compared (i.e. a predictor based on single sources, multiple sources, ensembles, or local ancestry-aware approaches)? Second, in whom is it being compared (i.e. ancestry populations, gradient of ancestry, admixed populations, or clinical subgroups? Third, under what data considerations (i.e. imbalanced sample sizes, discrepancy of LD references, limited variant sets, heterogeneous phenotypes and sparse validations)? Fourth, against which criteria (i.e. discrimination, calibration, equity gaps, uncertainty and computational feasibility)? Lastly, applying which safeguards (i.e. leakage detection, standardised protocols, transparent tuning and subgroup analysis)?

### Pragmatic baselines and current practice in diverse cohorts

Although multi-ancestry approaches are becoming more common, many applied settings remain constrained by limited discovery data in the target ancestry, limited independent validation data and restricted computational resources. In that context, clumping and thresholding (C + T) is useful as a transparent, fast and auditable baseline. Its practical value is that it provides a low-complexity reference point against which the added value of more specialised methods can be assessed when LD references, variant sets and tuning constraints are held constant.

Ongoing work in African and other underrepresented populations provides an example of how this practical application is achieved. Studies such as Kamiza et al. use accessible scoring strategies and ancestry-stratified reporting to show how performance varies across African regions and between ancestry groups [8, 9]. Such work should not be construed to mean that C + T will be optimal in a given multi-ancestry setting. Rather, they show why transparent baselines remain informative. C + T defines performance using the fewest possible assumptions, thereby setting the stage for comparisons against more specialist methods. if performance varies within Africa and between ancestry groups, then a method claiming superiority must be benchmarked with stratified reporting, LD-reference sensitivity and transparent tuning, otherwise apparent gains may merely reflect who was evaluated, which reference was used or how parameters were selected. Table 1 summarises the multi-ancestry PGS software tools discussed in this review, including their required inputs, supported population structures, SNP restrictions, LD reference requirements and mechanisms for cross-ancestry borrowing.

**Table 1:** Multi‑ancestry polygenic score software tools

Tool: year and citation	Category	Required inputs	Input pops (N)	SNP restrictions?	LD reference panel support	Details of Algorithm/cross- ancestry borrowing
PRS-CSx: 2022 [20]	Bayesian shrinkage (multi-ancestry continuous-shrinkage)	1. SUMSTAT; 2. LD REF (per ancestry); 3. VAL/TEST recommended for mixing weights	K (≥ 2)	Default: HapMap3 (~ 1.3 M) because author LD panels are HM3. Denser sets possible with custom LD	Requires ancestry-matched LD reference for each population. Authors provide precomputed 1000 Genomes Phase 3 (and UK Biobank) LD panels for HM3 via PRS-CSx downloads; users can supply custom LD computed from a matched reference cohort	Joint Bayesian regression on multi-ancestry GWAS summary statistics with a shared continuous-shrinkage prior, while modelling LD separately in each population. Outputs posterior SNP effects per ancestry; final PRS often formed by stacking ancestry-specific PRS with weights learned in a tuning set
BridgePRS: 2024 [21]	Bayesian shrinkage (two-stage Bayesian ridge bridging)	1. SUMSTAT (discovery + target) 2. LD REF (matched to target, optional discovery) 3. VAL/TEST recommended for tuning	2(auxiliary + target)	None inherent. Typically common, well-imputed SNPs (HM3 often used for comparability)	Uses LD information to model within-locus correlation in each ancestry. LD can be derived from external reference panels (e.g., 1000 Genomes) or from large matched cohorts (e.g., UK Biobank, subject to access)	Fits a ridge-style PRS in a well-powered discovery ancestry, then updates or reweights effects using a smaller target-ancestry GWAS. Sharing is controlled so that evidence is borrowed where effects transfer, but target-specific deviations are retained
SDPRX: 2023 [22]	Bayesian shrinkage (nonparametric mixture; Dirichlet-process)	1. SUMSTAT (2 pops) 2. LD REF (2 pops)	2	No hard restriction. Requires SNP overlap between SUMSTAT and LD REF (common SNPs often used for stable LD)	Requires ancestry-specific LD matrices (typically block-wise). Users compute LD from ancestry-matched reference genotypes (e.g., 1000 Genomes) or from in-sample data. These matrices are provided as inputs	Jointly models marginal GWAS effects from two populations while accounting for population-specific LD. A nonparametric prior allows SNPs to be null, population-specific, or shared (correlated) across ancestries, yielding posterior SNP weights per population
XPASS/XPASS + : 2021 [23]	Bayesian correlated-effects model	1. SUMSTAT (target + auxiliary) 2. LD REF (target + auxiliary)	2	No array restriction; requires harmonised SNP overlap and matched LD (common SNPs recommended)	Requires population-matched LD matrices for both target and auxiliary populations. LD can be computed from reference panels (e.g., 1000 Genomes) or a matched cohort; provided as input to the software	Models SNP effects in target and auxiliary populations jointly as correlated random variables, so borrowing is proportional to estimated cross-population genetic correlation. XPASS + adds a sparse set of large, population-specific fixed effects (selected by pruning-and-thresholding) to improve robustness when some loci do not transfer
MUSSEL: 2024 [24]	Bayesian shrinkage + super-learner ensemble	1. SUMSTAT (K pops) 2. LD REF (per pop) 3. VAL/TEST (tuning for super learner)	K (≥ 2)	Evaluated on HM3 + MEGA (~ 2 M). Other variant sets possible with matching LD (compute cost increases with density)	Uses ancestry-specific LD references for each training population. The paper uses 1000 Genomes genotype data to estimate LD. Users can compute LD from 1000 Genomes or other matched references and supply it to the pipeline	Constructs multiple ancestry-specific PRS (and combinations) and learns an optimal cross-ancestry ensemble in a tuning dataset using a super learner. This lets different ancestries and base predictors contribute in a data-driven way under sample-size imbalance
X-Wing: 2023 [25]	Bayesian PRS with local genetic correlation + functional annotation	1. SUMSTAT (target + auxiliary) 2. LD REF (both). Functional annotation (as specified by implementation)	2 (repeatable for multiple auxiliaries)	No hard restriction; commonly run on high-quality common SNPs with annotation coverage	Requires population-matched LD references for each population to estimate local genetic correlation and fit the Bayesian model. LD is typically derived from 1000 Genomes or similar reference panels; user-supplied	Identifies genomic regions with shared local genetic correlation between populations and uses this, together with functional annotation, to adapt shrinkage across the genome. Produces improved PRS and can combine PRS from multiple populations using summary statistics alone
CT-SLEB: 2023 [26]	Hybrid: clumping + thresholding + Empirical Bayes + stacking (super learning)	1. SUMSTAT (K pops) 2. LD REF (per pop) 3. VAL/TEST (for stacking)	K (≥ 2)	Often HM3 or HM3 + MEGA for stable LD and compute; broader sets possible with matching LD	Needs ancestry-specific LD references for clumping and LD-aware steps. LD is commonly computed from 1000 Genomes reference samples. Clumping typically uses PLINK with an ancestry-matched LD reference	Builds a target-ancestry C + T score, then applies Empirical Bayes reweighting informed by auxiliary-ancestry GWAS, and finally uses super learning to combine candidate scores. Borrowing is explicit and tunable, rather than assuming identical effects
TL-PRS; 2022 [27]	Transfer learning (machine learning). Gradient-descent based fine tuning	1. SUMSTAT or GENO + PHENO 2. VAL/TEST (for tuning/early stopping) 3. LD REF depends on how the base PRS is built	2 (source + target)	Inherits from base PRS and SNP overlap between source and target; no built-in restriction	No extra LD panel beyond what is required to construct the baseline PRS (e.g., PRS-CS, LDpred). Fine-tuning updates weights using target data	Starts from SNP weights trained in a well-powered source population and updates them in the target population using gradient-based optimisation to improve prediction fit
PROSPER: 2024 [28]	Regularised regression (Elastic Net) ensemble for multi-ancestry PRS	1. SUMSTAT (K pops) 2. LD REF 3. VAL/TEST (for tuning/combining models)	K (≥ 2)	Typically common SNP sets (HM3 or HM3 + MEGA) depending on available LD reference	Requires an LD reference to map summary statistics into a penalised regression framework. LD can be computed from ancestry-matched references (1000 Genomes/UK Biobank) and supplied by the user	Fits penalised regression style models (Elastic Net family) using summary statistics and LD, then ensembles models across penalties and ancestries to improve robustness when architectures differ and sample sizes are imbalanced
XP-BLUP: 2017 [29]	Mixed model/BLUP-based cross-population predictor	1. SUMSTAT 2. GENO + PHENO (target training); 3. PCs	≥ 2 (auxiliary + target)	No hard restriction; depends on genotyping/imputation and QC (often pruned/common SNPs)	Does not require an external LD panel. Uses genetic relationship matrices derived from the individual-level target genotypes; auxiliary GWAS informs SNP set and weighting.	Uses a trans-ethnic (auxiliary) GWAS to prioritise informative SNPs and then applies BLUP/mixed-model prediction in the target ancestry using individual-level training data, combining transferred signal with target-specific estimation.
XPRS: 2025 [30]	Biology aware PRS models	1. SUMSTAT + gene/region annotation 2. GENO optional (to compute per-individual contributions)	N/A (interpretation layer)	Inherits from input PRS file	No LD reference required for basic decomposition (unless optional LD-aware aggregation is used)	Explains an existing PRS by decomposing it into contributions from SNPs and aggregated genomic units (genes/regions), producing an audit trail for interpretation. It does not itself perform cross-ancestry borrowing
RICE: 2024 [31]	Rare + common variant integration (penalised/ensemble)	1. SUMSTAT 2. LD REF 3.Rare-variant/ Exome features (implementation- specific); 4.VAL/TEST	≥ 2	Combines common and rare variant sets. Not limited to HapMap3	No extra LD panel beyond what is required from component PRS methods	Learns shared risk via mixed-ancestry ensemble models. Captures risk from both common and rare variants across ancestries
GAUDI: 2024 [32]	Local-ancestry-aware PRS for admixed individuals	1. SUMSTAT 2. GENO 3. PHENO + 4. LAI	Admixed; mainly two-way LAI in practice	No inherent restriction; variant set and pruning depend on compute and LD within local-ancestry tracts	LD is modelled within local-ancestry tracts using ancestry-specific information. Local ancestry inference itself requires reference panels (often 1000 Genomes or matched references)	Accounts for local ancestry mosaics by modelling ancestry-differential effects across the genome and borrowing information across shared ancestry segments, improving prediction when global ancestry labels are insufficient
HAUDI: 2025 [33]	Local-ancestry-aware PRS for admixed individuals	1. GENO 2. PHENO 3. LAI 4. PCS 5. TEST/VAL	Admixed; multi-way (K ≥ 2) LAI supported	No restriction	No external LD panel required for fitting. Requires LAI inferred using reference	Re-parameterises GAUDI into a standard LASSO formulation to estimate ancestry-specific effects with penalisation that encourages sharing while allowing differences. Scalable beyond two-way admixture
DisPred: 2023 [34]	Deep learning (multi-ancestry representation disentanglement)	1. GENO + PHENO (+ ancestry labels or PCS, depending on implementation)	K ≥ 2	Not fixed; depends on model input representation and QC (often pruned/common SNPs)	No explicit LD reference panel is required; LD is implicitly present in genotype inputs. Careful design is needed to avoid confounding from population structure and covariates	Trains a neural network to separate ancestry-related genetic variation from trait signal so that prediction relies less on ancestry-specific structure and generalises better across populations
DiscoDivas: 2025 [35]	Genetic-distance-assisted PRS interpolation/combination across ancestry continuum	Component PRS files from multiple fine-tuned models; target-individual PCs; median PCs/ancestry coordinates for fine-tuning cohorts; optional shrinkage weights	≥ 2 fine-tuning populations/models; target ancestry treated continuously, including admixed individuals	No inherent SNP restriction; variant set determined by upstream PRS models	Does not directly model LD; LD handling inherited from upstream PRS construction method	Combines multiple fine-tuned PRS models using genetic distance in PC/ancestry-continuum space. Produces harmonised PRS where matched fine-tuning data are unavailable or underpowered
iPGS: 2023 [36]	Inclusive individual-level PGS training using penalised regression	Individual-level GENO + PHENO; covariates/PCs; ancestry-diverse training sample. Pre-trained weights can be applied to target GENO.	Not population-count based. Supports ancestry-diverse and admixed individuals across a continuum	No fixed SNP set. Variants depend on genotype QC and penalised-regression selection	No external LD panel required; LD captured implicitly from individual-level training genotypes	Fits one penalised regression model across ancestry-diverse individuals, estimating ancestry-shared effects rather than excluding admixed individuals or assigning discrete ancestry bins
SSLPRS: 2025 [37]	Sparse PRS (spike-and-slab LASSO; Bayesian variable selection)	1. SUMSTAT 2. LD REF 3. VAL/TEST optional for tuning	1 (run per ancestry; can then stack across ancestries)	No hard restriction; depends on LD reference and QC (often common SNPs)	Requires an LD reference matrix; can be derived from in-sample or external references (e.g., 1000 Genomes, UK Biobank) that match the target ancestry and variant set	Infers a sparse set of SNP effects by using a spike-and-slab lasso prior, accounting for LD. Not intrinsically multi-ancestry, but can be applied separately within each ancestry and combined using stacking/ensembling

Summary of software tools that can construct PGS in settings where discovery and target data span more than one genetic ancestry. For each tool, we report (i) any Single Nucleotide Polymorphism (SNP)-level restrictions imposed by the method, (ii) the number of input populations that can be modelled jointly, (iii) the minimum required inputs needed to run the software, and (iv) descriptions of LD reference panel support and the algorithmic mechanism for cross-ancestry borrowing

*SUMSTAT* GWAS summary statistics, *LD REF* LD reference panel/matrices, *GENO* genotypes, *PHENO* phenotype, *VAL/TEST* individual-level validation/tuning set, *LAI* local ancestry inference information, *PC*s genetic principal components

## Methodological frameworks for multi-ancestry PGS

**Fig. 1:**
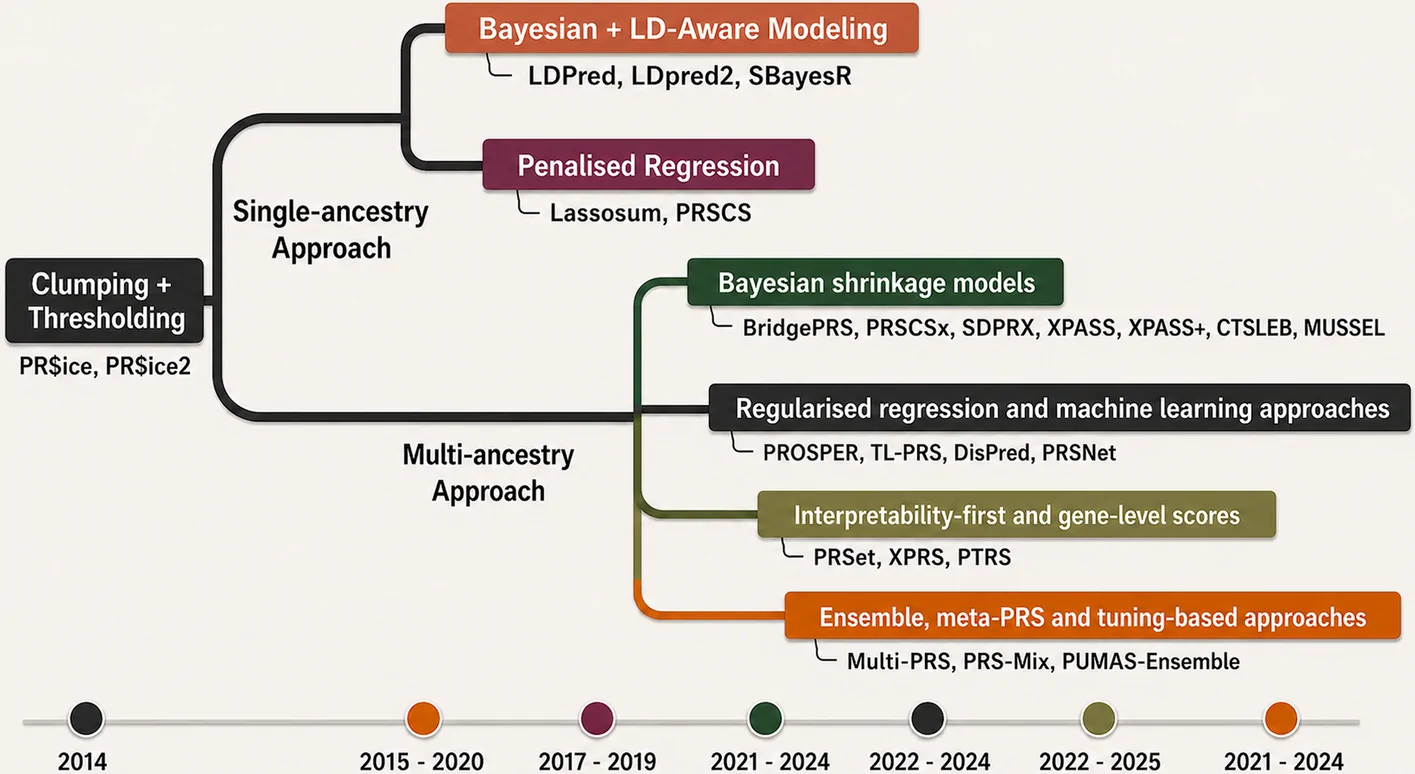
Schematic overview of PRS methodological families. This schematic summarises the major modelling families used to construct polygenic scores and their progression from early, single-ancestry pipelines to more recent multi-ancestry approaches. Each coloured box denotes a methodological family and lists representative implementations. The layout and connecting branches highlight broad conceptual relationships and shared modelling ingredients, not a strict historical lineage. Many approaches were developed in parallel and some could reasonably fit multiple categories. The tree begins with C + T methods, which select approximately independent SNPs and weight them using GWAS summary statistics. Subsequent single-ancestry approaches improved performance by modelling LD and stabilising noisy GWAS effect estimates through regularisation, including penalised regression, related shrinkage methods and Bayesian LD-aware regression frameworks. Multi-ancestry approaches explicitly target portability by incorporating cross-population structure. Bayesian trans-ancestry shrinkage models couple effect distributions across ancestries. Regularised regression and machine-learning methods optimise predictive weights with ancestry-aware tuning or adaptation. Ensemble, meta-PGS and tuning-based approaches combine or tune multiple scores within the target cohort. The timeline reflects approximate publication windows for each family. Branch lengths are conceptual and are not intended to represent methodological distance in a quantitative way

**Fig. 2:**
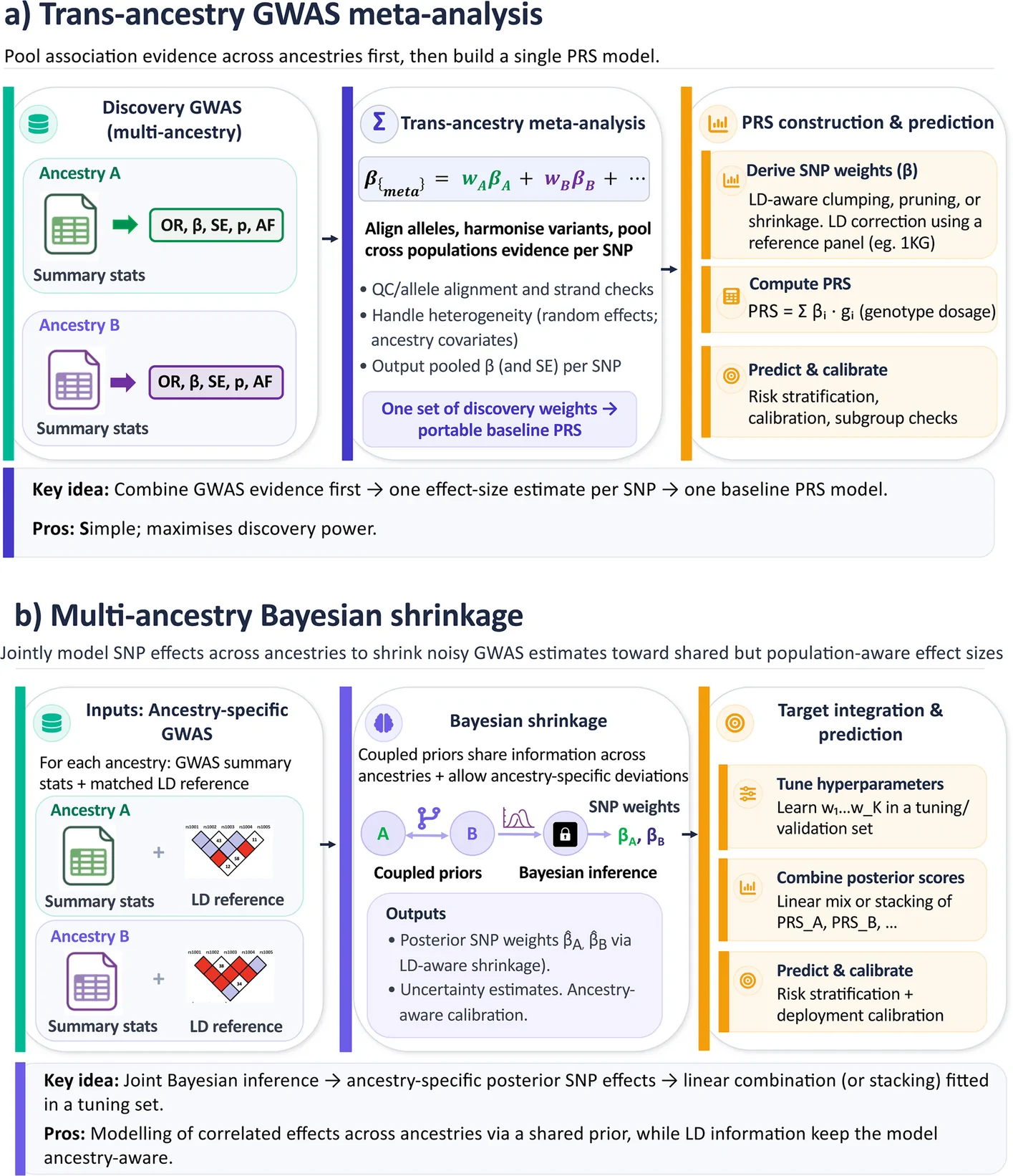
Generalised strategies for building polygenic scores across ancestries. **a** Trans-ancestry GWAS meta-analysis first pools ancestry-specific summary statistics to obtain a single per-SNP effect estimate, which is then used to construct one LD-adjusted PGS. This strategy can be implemented through standard or LD-aware single source methods after multi-ancestry meta-analysis. Examples of this meta-analysis strategy include multi-ancestry GWAS meta-analysis followed by standard PGS construction tools such as PRSice2, LDpred2, PRS-CS, DBSLMM or SBayesRC. Related score-level integration strategies include MultiPRS, PRS-Mix, SBayesRC-multi and PUMAS/PUMAS-Ensemble. **b** Illustrates Bayesian cross-ancestry borrowing strategies. In joint models such as PRS-CSx, SDPRX and XPASS, ancestry-specific summary statistics and LD references are modelled together through shared priors (PRS-CSx), covariance structures (XPASS) or SNP-level sharing states (SDPRX). BridgePRS follows a related but sequential route whereby it first learns a Bayesian ridge posterior in a well-powered source ancestry and then uses that posterior as an informative prior for the target-ancestry analysis, preserving locus-wide LD structure before final tuning or model selection. The locked black box in panel (**b**) denotes that the Bayesian inference central to each method is opaque to non-specialist users. Further insight into such methods is thus provided in the Additional Information 1

**Fig. 3:**
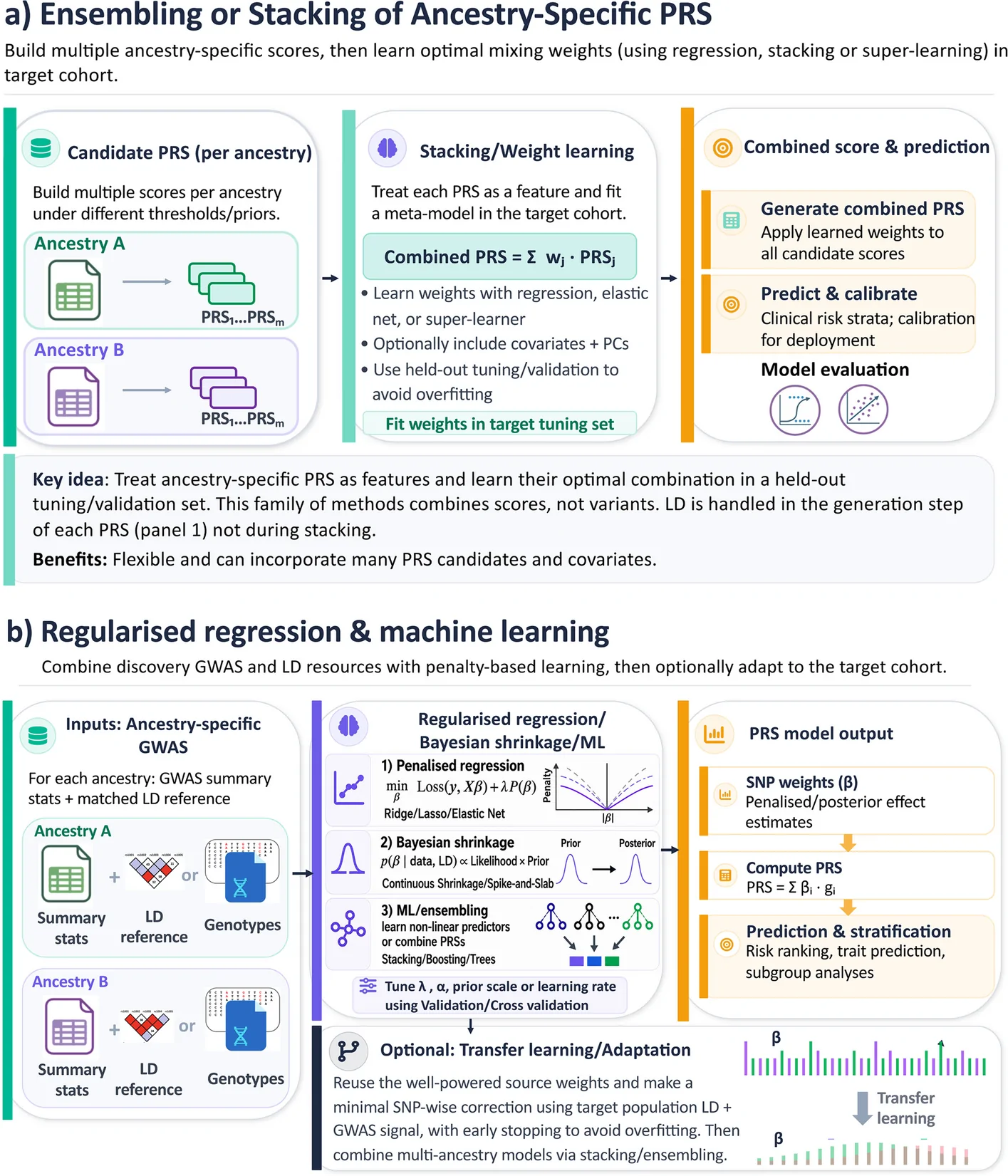
Rule-based and machine-learned combinations for multi-ancestry polygenic scoring. **a** Ensembling and stacking build multiple ancestry-specific PGS candidates and combine them either by simple averaging, fixed rule-based weights such as GWAS sample size, or learned mixing weights in a target tuning cohort, treating each PGS as a feature. The fitting step often uses supervised learning such as linear or logistic regression, or elastic net. **b** Regularised regression and machine-learning approaches jointly learn or adapt SNP weights from ancestry-specific GWAS, ancestry-matched LD references or individual-level genotypes, using penalisation, shrinkage or transfer-learning adaptation before scoring and performance evaluation

Early PGS methods prioritised speed and simplicity [38]. Current methods, developed over the last decade, focus on modelling LD explicitly and use shrinkage or other forms of regularisation to avoid overfitting to noisy marginal signals from GWAS [5, 39, 40]. Multi-ancestry PGS methods can be grouped into broad methodological families that share the aim of improving prediction across diverse ancestries, but differ in how they model LD, regularise effect sizes and share information across populations (Fig. 1). Figure 2a shows the framework behind meta-analysis approaches which combine GWAS summary statistics from various ancestries before computing PGS using conventional or LD-aware PGS softwares. Figure 2b contrasts this with Bayesian trans-ancestry strategies that jointly model ancestry-specific summary statistics and LD references. Figure 3a displays ensembling/stacking frameworks, which combine individual ancestry-specific PGS, whereas Fig. 3b shows regularised, machine-learning or transfer-learning approaches that learn or adapt weights under explicit tuning. Implicit in most multi-ancestry approaches is the assumption that there is overlap of genetic architecture across ancestries. This proposition makes information sharing sensible. The most successful approaches make effect-sharing conditional, by borrowing where the data support concordance, while preserving room for ancestry-specific departures [20, 21].

## Meta-analysis and source integration approaches

Figure 2a represents the most direct route to multi-ancestry score construction and involves combining GWAS summary statistics across ancestries first, then constructing a single score from the pooled discovery signal. This is often less a dedicated PGS algorithm than a benchmark-relevant workflow. For example, trans-ancestry meta-analysis or meta-regression can generate a single set of effect estimates, which can then be used by standard PGS builders such as PRSice2, LDpred2, PRS-CS, DBSLMM or SBayesRC. Its attraction is practical, as all discovery samples contribute to one summary statistics file and familiar tools can be applied without major changes. The main limitation of the approach is that a single meta-analytic effect estimate can obscure ancestry-specific tagging, LD differences and true effect heterogeneity. Related strategies therefore integrate information later, at the score level. MultiPRS combines scores trained in a large source population with scores trained in a smaller target population; PRS-Mix and SBayesRC-multi learn mixtures of ancestry or method-specific predictors and PUMAS/PUMAS-Ensemble use summary statistics to tune and combine candidate scores when individual-level validation data are limited [14, 41–44]. The implication for benchmarking is that gains from larger multi-ancestry discovery sample size should be separated from gains due to ancestry-aware modelling or score-level weighting. Failure to do this means that improved performance after pooling may be mistaken for evidence that a modelling framework has solved portability.

## Bayesian shrinkage models

Bayesian multi-ancestry PGS methods estimate the contribution of genetic variants to a trait or disease by analysing data from multiple populations, using a probabilistic framework that shares evidence across groups while still allowing for population-specific divergence (Fig. 2b). They do this using shared priors (assumptions about how similar effects tend to be [20]), hierarchical variance components (the decomposition of shared and population-specific variation [21]) or cross-population covariance structures (explicit modelling of the strength with which effects are shared across populations [22, 23]). In all tools, the power of a larger GWAS can stabilise effect estimation in a smaller, ancestry-targeted GWAS. Updated estimates of association for each variant are produced after accounting for population-specific correlation among variants, using LD information derived from ancestry-matched reference panels.

PRS-CSx uses continuous shrinkage to pull most variant effects towards zero while allowing a minority to remain non-zero when supported by the data. Because shrinkage strength is shared across ancestries, information from larger GWAS, often European, can help identify variants likely to carry signal in a smaller (non-European) target GWAS. The method produces ancestry-specific Single Nucleotide Variant (SNP) weights, constructs ancestry-specific scores and then learns an optimal linear combination of these predictors in a regression model. Cross-ancestry borrowing is therefore data-driven rather than imposed [20].

BridgePRS pursues a similar goal using a two-stage Bayesian ridge strategy. It first uses a large discovery GWAS to estimate source population SNP effects, then treats these estimates as an informative prior that is updated using a smaller, ancestry-matched target GWAS [21]. The use of ridge shrinkage by BridgePRS allows retention of multiple correlated variants within a locus; spreading weight across many proxies that may tag the same underlying causal signal. This is a desirable feature when the true causal variant is missing, poorly imputed or not directly typed. The contrast between PRS-CSx and BridgePRS raises the question of which scenarios do methods that localise signal more strongly outperform methods that hedge by retaining correlated proxies under uncertainty.

A related class of trans-ancestry Bayesian PGS methods regulates information-sharing according to the inferred similarity of genetic effects across populations. In XPASS and its individual-level analogue XPA, SNP effects in the target and larger auxiliary populations are modelled jointly with a covariance term that reflects trans-ancestry genetic correlation. Therefore, the amount of information borrowed from the large study is estimated from the data itself rather than prespecified. XPASS + extends the model by including some ancestry-specific effects, chosen through pruning and thresholding, in a shared polygenic background. This improves robustness when some loci do not transfer cleanly across ancestries [23]. SDPRX also incorporates ancestry-specific LD, applies additional shrinkage to reduce inflation from LD-reference mismatch, and uses priors that allow SNPs to be null in both populations, active in one population only, or shared with correlation [22]. Further details of the described methods are provided in Additional Information 1.

### Implications for benchmarking

Trans-ancestry Bayesian approaches should be evaluated in the context of variation in sample size of target GWAS, genetic correlation between the populations, and LD reference alignment. The relative strength of Bayesian approaches may hinge on how differing methods value sparse signal localisation, robust tagging through correlated proxies, or accurate modelling of ancestry-specific LD and effect heterogeneity.

## Regularised regression, transfer learning and deep models

Regularised regression methods control overfitting in high-dimensional genetic prediction by adding penalties that reduce model complexity. The resulting shrinkage pulls weaker or redundant predictors toward zero while retaining stronger signals (Fig. 3b). The approach is computationally tractable and adaptable to multi-ancestry settings, especially when combined with ancestry-aware tuning or transfer learning. For example, the PROSPER method utilises an elastic net ensemble which combines models for different values of the penalty parameters and ancestries. The aim is to maintain consistent performance across varying genetic architecture and training data size [28]. Transfer learning reuses knowledge learned in one setting to improve prediction in a related setting, rather than building a new model from scratch. In TL-PRS, SNP weights from a model trained in a large GWAS are iteratively fine-tuned using target-ancestry evidence [28].

Most PGS methods implicitly assume additive SNP effects, so that each score is a weighted sum of allele dosages. Other higher order effects such as non-additivity, interactions or complicated haplotypic structures are often not considered in standard PGS approaches. Machine learning techniques overcome this limitation by allowing non-linear relationships between genotype and phenotype. This comes at a cost: because genotype data are structured by LD, population history and technical artefacts, flexible models can learn ancestry-correlated patterns that predict phenotype in the training data but fail to transfer across populations [45–47]. The DisPred model learns a deep neural network to predict a trait but attempts to ignore ancestral genetic structures to obtain better transferability and focus on biological signals [34]. PRS-Net uses graph neural networks to group variant signals into gene-level summaries and share information across connected genes, supporting biological interpretation as well as prediction [48]. These approaches address two limitations of standard scores: their limited capacity to model structural complexity and limited biological interpretability. However, added flexibility introduces additional degrees of freedom and greater sensitivity to training design, covariate adjustment, LD specification and hyperparameter tuning. It remains an empirical question when machine-learning PGS methods outperform strong linear baselines after LD, confounding, tuning and leakage are controlled [49].

### Implications for benchmarking

Regularised and machine learning methods must be benchmarked under identical hyperparameter selection criteria, validation designs, and control for sample leakage. Benchmarks must state whether performance gains are maintained under equal tuning budget, covariates, LD references, and size of target ancestry validation samples.

## Ensemble and meta-learning PGS approaches

If the discovery GWAS is ancestrally mismatched to the target cohort, or the target ancestry GWAS is small, apparent gaps between PGS methods can be driven as much by tuning and combination as by the underlying statistical model. One practical approach is weighted stacking [14]. This method involves generating at least two ancestry-based scores, preferably through the same standardised scoring pipeline, after which, mixing weight estimation is performed using an independent target validation set. Regression analysis utilising the score together with the standard covariates (see Fig. 3a) is performed. The obtained score represents a compromise between discovery sample statistical power and target ancestry compatibility [14]. Advanced stacking models can include several scores to find the best mixture for each particular phenotype and target sample; PRS-Mix reflects the observation that no single construction strategy performs best across all settings [41]. Stacking also adapts tools not designed for multi-ancestry deployment, as Zheng et al. demonstrated by applying SBayesRC separately within each ancestry and combining ancestry-specific outputs using target ancestry tuning data [44]. PUMAS and PUMAS-Ensemble reduce the reliance on individual-level validation data, through tuning and aggregation based on summary statistics [42, 43]. CT-SLEB uses shrinkage combined with the super-learner technique, whereas MUSSEL extends this hybrid strategy [24, 26].

### Implications for benchmarking

Ensemble and tuning-based methods require benchmarks that separate gains from the base scoring method, gains from the tuning procedure and gains from the stacking or weighting layer. Reports should specify candidate score libraries, validation data, weight-estimation procedures and whether the same tuning opportunities were available to all methods.

## Community infrastructure for scalable and reproducible PGS benchmarking

The translational applications of PGS benchmarking depends on the existence of common infrastructure, which includes curated score definitions, reproducible pipelines and reporting standards that make results comparable across studies. The PGS Catalogue curates standardised scores according to FAIR (i.e. Findable, Accessible, Interoperable and Reusable) principles, allowing for their reuse and evaluation in different settings [19, 50]. Polygenic Risk Score Reporting Standards (PRS-RS) complements this by specifying minimum reporting requirements for study design, phenotyping, model construction, validation and interpretation [15]. Services such as Imputation Server PGS integrate ancestry estimation and score calculation into imputation workflows, which helps to lower barriers for non-specialists [51]. A growing set of tools can now standardise larger parts of the workflow (Table 1). GenoPred implements automated harmonisation, LD-aware modeling, scoring and reporting [16, 52, 53]. PRS-UP caters to underrepresented groups where validation data is limited [54]. PGSFusion provides a web server that enables parallel running of various methods along with reporting [55]. STREAM-PRS and PRSpipe implement modular operations and facilitate comparison between biobanks [42, 43]. UKB-pret compares scores for different ancestry in the UK Biobank [56]. Consortium-level initiatives such as PRIMED provide an organisational foundation for multi-site harmonisation and shared analysis environments [57].

## Common design elements in benchmarking studies

The rapid expansion of multi-ancestry PGS methods (Figs. 2 and 3) has shifted the fieldwide bottleneck from the supply of new algorithms to the development of rigorous frameworks for their systematic appraisal [16, 42, 54]. As GWAS become larger and more diverse, the key question is whether scores generalise reliably at the point of use. Beyond quantifying transferability in broad terms, there is a need to define the subgroups and individuals for whom scores work and also fail. The uncertainty in ranking, outcome prediction and absolute risk must also be reported [10, 58, 59]. The ability to resolve such questions depends as much on choices made during the benchmarking process as on the specific evaluation method.

### Hidden degrees of freedom in benchmarking pipelines

Even small variations, including phenotype definitions, covariate adjustment, LD reference choice, variant filtering, and parameter tuning techniques, may affect assessments of method performance and ranking by adding noise. This does not mean that every workflow difference invalidates a benchmarking study; within a well-controlled study, comparisons can be meaningful if the same harmonisation, quality control and evaluation rules are applied consistently across methods. However, there will be limitations in the ability to generalise across multiple studies or due to the lack of information about specific pipeline choices. Thus, benchmarking must be viewed as a problem of experimental design and major decisions documented to ensure that any observed differences do not stem from cohort characteristics or pipeline specifics rather than methods. Large biobanks have demonstrated the importance of properly handling such issues, given that between-cohort variability can be comparable to (and even exceed) variation between methods [53].

### Populations, traits and performance metrics

Recent benchmarking studies routinely include African, East Asian and South Asian ancestry groups alongside European ancestry, but representation remains uneven and ancestry assignment is not always standardised. Admixed populations provide a distinct stress test because a single global ancestry label can conceal many different mosaics of local ancestry across the genome, complicating LD modelling and limiting methods that rely on a single ancestry-matched reference panel. Proper evaluation therefore requires local-ancestry-aware approaches, yet these are absent from many multi-ancestry PGS tools (Table 2) [32, 61]. More broadly, coarse ancestry labels can obscure ancestry gradients and genetic distances that influence portability within and between groups [58]. These choices hence affect how performance is estimated, interpreted and reported for a substantial proportion of the global population.

**Table 2:** Available benchmarking resources for multi‑ancestry polygenic scores

Benchmarking tool [Citation number]	Publication year	Workflow language/interface	Included PRS tools	Features/modules	Scope/populations	Key findings
PRS-UP/PRSUP [54]	2022	Python/Jupyter-based command-line framework. Repository provides tutorials and Conda environment. Some methods require R packages such as bigsnpr/susieR	**Combines 13 methods:** **Single-ancestry** C + T Double-weighted PRS; LDpred LDpred2 lassosum SBayesS BayesR SBayesR MegaPRS **Multi-ancestry** PRS-CSx PolyFun PolyPred PolyPred +	Benchmarks PRS transfer to under-represented populations using two strategies: (i) meta-population analysis with combined individual-level genotypes and (ii) multi-population analysis using population-specific summary statistics. Supports simulated and real-data evaluations	Designed for settings where the target population has limited genomic data. Demonstrated using Vietnamese data, with transfer from larger reference populations including EUR, EAS/JPN, SAS and AFR	Best method/source population is context-dependent; cross-population borrowing can improve prediction but is not uniformly beneficial
GenoPred [16]	2024	Snakemake command-line pipeline with Conda environments; scalable on HPC/Cloud	**Combines 8 methods:** **Single-ancestry** single-trait C + T lassosum DBSLMM LDpred2 SBayesR MegaPRS QuickPRS PRS-CS **Multi-ancestry:** PRS-CSx, TL-PRS, X-Wing	End-to-end GWAS and target-genotype QC, ancestry inference, PRS generation, pseudovalidation, target scoring and reference-standardised score reporting **PRS models** are optimised using method-specific tuning strategies: **Single-ancestry PRS methods** use pseudovalidation to select hyperparameters directly from summary statistics **Multi-ancestry PRS methods** include TL-PRS, which adapts an existing PRS model using summary statistics from another population. **LEOPARD + QuickPRS** combine population-specific PRS models using analytically derived linear weights **TL-PRS** and **LEOPARD + QuickPRS** are applied only to user-selected single-source PRS models that have been pre-optimized via pseudovalidation	General purpose PRS pipeline for GWAS/target data from populations represented in the reference panel. Supports diverse ancestry studies via 1 KG/HGDP-based ancestry inference and score standardisation	Improves reproducibility and comparability by standardising preprocessing, ancestry assignment, PRS generation and scoring; UK Biobank application demonstrates scalability and practical performance. Runtime breakdowns are provided to reveal practical compute trade-offs across methods
PGSFusion [55]	2025	Web server: pgsfusion.net Backend code in R	**Combines** **17** **methods**: **Single-ancestry** C + T DBSLMM DBSLMM-auto DBSLMM-lmm lassosum2 LDpred2 LDpred2-auto MegaPRS-BayesR PRS-CS PRS-CS-auto SDPR **Multi-trait** mtPGS **Annotation-based** AnnoPred SBayesRC **Multi-ancestry** PRS-CSx SDPRX XPASS	Automated GWAS summary-statistic parsing; method selection. Ready-to-use reference panels. UK Biobank validation resources for 201 traits; performance evaluation; joint/covariate analysis. Downloadable plots/results	Accessible PGS construction and comparison for non-specialists; supports EUR, EAS and AFR reference resources, with cross-ancestry applications demonstrated	Lowers technical barriers to running many PGS methods, standardises input handling and reporting and enables practical comparison of methods, prediction performance, computational cost and downstream epidemiological associations
STREAM-PRS [52]	2025	Bash-driven command-line workflow. Wraps R/Python PRS tools; Conda environment provided.	**Combines 5 methods:** **Single-ancestry** PRSice2 PRS-CS LDpred2; lassosum; lassosum2	Four-stage workflow: GWAS QC/formatting; multi-tool score calculation with parameter grids; PC correction and standardisation; best-score selection by predictive performance	Within-study PRS construction, tuning and comparison. Demonstrated mainly in European/Non-Finnish European -to-UKB settings for IBD	Standardises multi-tool PRS comparison. Highlights how standardisation and variant set overlap (e.g., restriction to HapMap3 SNPs) can affect portability and comparability. best method is data-dependent; IBD example favoured lassosum
PGS-hub [60]	2026	Web-accessible benchmarking platform: https://ngdc.cncb.ac.cn/pgs-hub/ Offline workflow also available	**Combines 10 methods** **Single-ancestry** C + T Lassosum2 LDpred2 MegaPRS PRS-CS SBayesR SBLUP SDPR **Multi-ancestry methods** PRS-CSx X-Wing	Automated summary statistic formatting; method-specific parameters; built-in 1000G LD panels for EUR/AFR/EAS/SAS; custom LD upload; evaluates accuracy, risk stratification, SNP number, compute cost and LD-panel size	Designed for harmonised comparison of single and multi-ancestry PGS methods; benchmarked in UK Biobank European and African ancestry samples	LDpred2 performed strongly among single-ancestry methods; LDpred2-multi outperformed PRS-CSx and X-Wing in multi-ancestry analyses. LD reference size improved performance below ~ 1,000 samples and plateaued beyond ~ 5,000

Resources that support the construction, tuning, and head-to-head evaluation of polygenic scores across diverse ancestry groups. For each framework, we summarise the delivery modality and the PRS construction methods implemented. Where available, we also summarise the evaluation outputs emphasised by the original publication. Capabilities are reported as described in the cited references

Benchmarking studies often prioritise traits with abundant GWAS data, usually combining continuous phenotypes such as height, body mass index and lipids with binary outcomes such as type 2 diabetes and coronary artery disease. This choice is not neutral. Trait architecture describes how genetic effects are distributed across the genome. Some traits are highly polygenic, with many small effects, while others have sparser architectures, larger locus-specific effects, more environmental involvement, or variable measurement. Methods can therefore behave differently across height, lipid traits, autoimmune disease, psychiatric disorders and cancers. Where possible, benchmarks should incorporate a wider variety of trait architectures and should also avoid claiming overall superiority of one approach over another, based on narrow panels of selected traits [62–64]. The outcome type also shapes evaluation. Continuous outcomes are summarised and assessed using R^2^, whereas binary outcomes may be assessed using either Nagelkerke’s R^2^ or AUC. Calibration, a measure of concordance between predictions and outcomes, is less consistently reported, despite being central to clinical interpretation. Finally, equity assessments (comparing performance, calibration, or error across ancestry or other meaningful subgroups) are often neglected. Rigorous benchmarks should assess discrimination, calibration and equity by ancestry, and other relevant subgroups [65, 66]. Use of mean AUC or R^2^ within large ancestral strata can mask two key considerations. The first is that performance tends to decrease gradually with increasing distance from ancestral origin, with the worst performance occurring at the extremes [58]. Secondly, equity should be evaluated relative to absolute performance, because true equity cannot occur if discrimination is poor and/or miscalibrated for all subgroups. Simple disparity measures, such as minority-to-majority ratios (e.g. AUC for African-ancestry divided by AUC for Europeans (or the equivalent for R^2^), or alternatively, improvement over a single-ancestry baseline) may help make progress easier to track.

### Harmonisation, QC and LD specification

Inputs, harmonisation and LD specification are frequent sources of hidden variation in PRS benchmarking studies. Within a single well-controlled benchmarking study, consistent quality control, harmonisation, LD-reference selection and variant filtering can support fair comparisons made across different methods. However, these choices are not neutral; rankings may vary under alternative, equally defensible study designs and workflow choices, unless those choices are transparently reported. Most studies begin with GWAS summary statistics, but converting these heterogeneous files into a harmonised, PRS-ready variant set involves many steps that can be implemented differently and may be hard to reconstruct from published descriptions. The minimum requirements for harmonisation are as follows: (i) genome builds should be consistent (e.g., GRCh37 vs. GRCh38) with liftover used if necessary (ii) variant IDs should be unique (rsID and/or chromosome location with alleles) and (iii) allelic alignment needs to be accurate to guarantee that the effect allele in the GWAS study corresponds to the coded allele in the target genotypes. Frequently, issues with strand designation can lead to errors, particularly for palindromic SNPs such as A/T and C/G, where strands can be flipped and the SNPs become ambiguous. This often necessitates excluding SNPs or adjusting allele frequencies. The problem is further complicated when allele frequency distributions differ between the discovery GWAS and the target cohort, for example across ancestry groups, because frequency-based checks used to resolve strand ambiguity in palindromic SNPs may be unreliable when the reference and target populations have different allele frequencies [67]. Benchmarking studies also differ in how they handle multi-allelic sites, insertion/deletion events (indels), duplicated rsIDs and variants with inconsistent alleles across cohorts or reference files [15].

Variant filtering choices can compound these issues. Most studies apply thresholds to imputation quality (e.g. INFO/R^2^) and minor allele frequency (MAF). However, the consequences may not be similar across ancestries, as the performance of imputation and the distribution of minor allele frequency can vary across populations and by reference panel. The use of a fixed INFO or MAF can remove a larger fraction of variants in some cohorts compared to others, thus turning a methods comparison into a comparison of effective variant sets. This is especially so when using data from multiple ancestries that have imputation references and genotyping arrays that differ across the cohorts. The choice of LD reference set used may influence predictive accuracy and comparisons of performance across populations. European reference panels are often larger and better matched to European cohorts, whereas African and admixed cohorts may rely on smaller or less representative references (or imperfect single-ancestry proxies). As a result, LD misspecification can disproportionately affect non-European targets, by producing less stable LD estimates that widen cross-ancestry performance gaps, for reasons that are partly methodological and hence potentially avoidable [11].

Variant selection strategies vary across design frameworks. For instance, some Bayesian shrinkage methods use HapMap3 single nucleotide polymorphism (about one million common, well-imputed variants) by default to stabilise linkage disequilibrium estimation and to save computing time [20, 39]. While this improves reproducibility across studies it can also exclude informative variants and may favour methods optimised for common variant tagging [20, 39] while downweighting the performance of tools designed to accommodate denser variant sets, lower frequency variants and ancestry-enriched variation. This point is especially pertinent to populations of African origin, which harbour the greatest levels of human genetic diversity and have a higher burden of rare and population-specific variants compared to non-African populations [6].

### Benchmark integrity and auditability

Collective variation in phenotype definition, covariate adjustment, ancestry assignment, LD-reference selection, variant filtering, harmonisation procedures and tuning strategy makes clear that benchmarking should be defined and reported with the stringency of an experimental protocol. At a minimum, studies should report their harmonisation rules (including their handling of variant ambiguity), variant filtering rules (including their specific attrition rates) and their LD reference specifications (ancestry, etc.). They should also detail the specific steps taken or safeguards imposed to prevent information leakage between discovery and evaluation datasets. Even small amounts of sample overlap can inflate performance estimates. Relatedness between training and test samples can similarly introduce optimistic bias. Tools and guidance to detect overlap exist, but are under-utilised [1, 68]. Finally, whether a benchmark can be trusted and reused depends on reproducibility. In this regard open code, containerised environments, pinned software versions and documented random seeds are increasingly expected [69–71]. From a benchmarking perspective, the computational setup is an important aspect of reproducibility [66, 72]. Runtime, peak memory and hardware assumptions can help to determine whether a method is feasible to run, or can be executed at biobank scale, rerun across multiple cohorts and audited by independent groups. They also determine which methods are practical to run in resource-constrained settings. Without this transparency, benchmarks may end up favouring methods that are easiest to execute in the benchmark setting, rather than those that are best aligned with the populations, phenotypes and intended deployment contexts that matter in practice [73].

### Simulations as controlled benchmarking experiments

Even when reporting and reproducibility standards are met, purely empirical comparisons can remain sensitive to the idiosyncrasies of any single cohort or deployment context. Simulations therefore serve a complementary role, not as a replacement for empirical multi-cohort evaluations, but as targeted stress tests in which the ground truth is already known [54, 74]. Because no simulation can fully capture the ascertainment, phenotype definition, environmental context, and technical artefacts of real cohorts, simulation should be used to diagnose method behaviour under controlled assumptions, rather than to define methodological hierarchies absolutely. Multi-ancestry simulations are most useful when they vary the parameters that determine portability, such as allele frequency spectra, LD structure, heritability, polygenicity, effect-size distribution, cross-population genetic correlation, genotype or imputation quality and sample size imbalance. Simulation methods that create phenotypes by drawing upon real genotypes, preserve realistic LD pattern and genotyping artefacts and are often more insightful than fully simulated genomes. Simulations must make their assumptions about the trait architecture explicit. The LDSC method correlates GWAS signals with the degree to which the SNP is correlated with the surrounding SNPs through LD [62]; in contrast, the LDAK-like models allow for the predicted effect of the SNPs to be dependent on LD and minor allele frequency [62, 63]. Different algorithms will perform differently in these two settings, and hence benchmarking scenarios could consider exploring both. Environmental variance will also play a role, since different measurement errors or environments will give rise to different SNP heritabilities even for identical causal effects [64, 75]. Admixture should be treated as a headline scenario rather than an edge case, using tools such as msprime, SLiM or HAPNEST to model demographic histories and local ancestry [74, 76, 77]. To address concerns that large shared datasets may be impractical or sensitive, simulation resources can be shared as scripts, parameter sets, random seeds, reference descriptions and evaluation code, allowing datasets to be regenerated where feasible.

## Future directions in multi-ancestry PGS benchmarking

### Multi-way admixture as the next frontier

Tools such as Tractor allow ancestry-specific association mapping in admixed cohorts by first using local ancestry inference, often from tools such as RFMix or close derivatives, to assign ancestry along each chromosomal segment [78]. Unlike classic admixture mapping, which tests whether local ancestry dosage at a region is associated with a trait, Tractor breaks down genotype dose by local ancestries and estimates the ancestry-specific effect of each SNP, thereby showing that discoveries and effect estimation may depend on tract (and not global) ancestry [78]. A small but growing set of PGS methods now operationalise this idea for prediction. GAUDI models SNP effects that vary across ancestry segments and borrows information across genomic segments sharing the same ancestry background [32]. slaPRS constructs population-specific scores in local windows and stacks them in a local-ancestry-informed manner [61]. DiscoDivas moves away from discrete ancestry assignment by interpolating predictor weights along an ancestry continuum [35]. Complementing tract-aware predictors, CalPred addresses a related bottleneck by using contextual information, including genetic ancestry and other covariates, to deliver calibrated risk estimates and context-aware prediction intervals, making uncertainty explicit when performance varies across groups or in admixed individuals [10].

However, most tract-aware predictors still treat inferred local ancestry as fixed and are either computationally demanding or primarily evaluated in two-way admixture settings. HAUDI extends GAUDI-style modelling towards faster computation and multi-way admixture [79]. Future work should invest in scalable multi-way admixture models, better-matched source reference panels for phasing and local ancestry inference, and methods that account for admixture-induced LD, genetic correlation between ancestries and uncertainty in inferred tracts rather than relying on fixed population labels.

### Building intelligent benchmarking frameworks

The PGS literature is saturated with new methods, yet the evidence base for deciding when to trust them remains immature. A major milestone for future benchmarking will be the shift towards intelligent automation of benchmark design choices, integrated with continuous learning capabilities. Benchmarking requires balancing of several competing goals, including discrimination, calibration, equity gaps and computational feasibility, rather than maximising a single metric in isolation. Automation can search design choices to identify options that meet pre-specified criteria while creating an audit trail that allows every decision to be inspected. Some elements already exist in current practice, including summary statistics based tuning and ensembling to reduce reliance on large validation datasets [43] and reproducibility-oriented pipelines that standardise harmonisation and evaluation across cohorts [16]. One route to automated benchmarking is to decompose optimisation into auditable stages: first select a tuned score using a declared rule, then apply a separate cross-population adaptation or weighting step. The latest GenoPred implementation illustrates this modular logic by using pseudo-validation for within-method tuning, followed by multi-source approaches such as TL-PRS fine-tuning or LEOPARD with QuickPRS linear weighting [80].

A desirable endpoint is a living benchmarking system that is version-controlled, containerised and continually updated as methods and datasets grow. The architecture of such a system would include linking empirical cohorts with simulation suites, while allowing for automated tuning under explicitly-stated computational budgets (in terms of CPU core-hours used per trait per cohort, preset maximum runtimes and memory usage, maximum number of hyperparameter configurations, and cloud compute costs per run). The system would yield scorecards for discrimination, calibration, outputs stratified by equity, and compute cost along with the uncertainty associated with each of these. Each run would also produce a machine-readable manifest describing inputs, parameters, LD references, ancestry definitions and data splits, enabling exact reproduction and tracking of method changes over time. Automated approaches have the potential to support expert judgment rather than replace it, given that optimisation processes can over-fit data or introduce bias if objectives are poorly specified. It is therefore necessary to include such guardrails as holdout datasets that are locked for any tuning, subgroup analysis, and ancestral group calibration. With proper safeguards in place, updated benchmarking becomes a way to convert a one-off comparison into evidence, thereby accelerating the maturation of equitable PGS.

## Conclusion

The next five years will be defined not by a quest for a single superior algorithm, but rather, on the ability of the field to tailor polygenic prediction to more complex biological and clinical scenarios. Three factors will guide this transition. The first is that future discovery efforts are becoming more ancestrally heterogeneous; improving mean portability while uncovering more granular structure within ancestral groups. In order to accommodate this, benchmarking standards will therefore need to incorporate the evaluation of ancestry along a continuum. The second is that local-ancestry-aware prediction will become the norm for admixed populations, creating urgent demand for scalable multi-way admixture models that are able to quantify tract-level uncertainty and account for admixture-induced LD. Finally, the challenge posed by context shift will continue to play a central role because differences in phenotype definitions, ascertainment, and environments are currently among the major determinants of absolute-risk misclassification, even in the absence of rank-order changes [11, 12]. Together, these shifts will move the field forward, from asking whether a score is portable on average, to asking whether its reliability can be demonstrated across heterogeneous populations and settings. The next generation of benchmarking studies must therefore test whether uncertainty is quantified, calibration is preserved, and the limits of reliable use are known before deployment. If this process can become embedded into software design, consortium efforts and reporting standards, then polygenic prediction-making will be better placed to serve the translational goals of genomic medicine. Without such a framework, then increasingly sophisticated models may continue to make clinical claims that remain valid only within the narrow range of settings in which they were tested.

## Supplementary Information

Additional file 1. The guiding logic and intuition of Bayesian multi-ancestry polygenic risk score methods. Supplementary methodological text providing a method-by-method explanation of seven Bayesian approaches for multi-ancestry polygenic risk score construction: PRS-CSx, SDPRX, BridgePRS, JointPRS, MUSSEL, XPASS and X-Wing. The file explains the shared LD-aware Bayesian framework, how each method models ancestry-specific linkage disequilibrium, how information is borrowed across ancestries, the role of tuning or validation data, and how resulting SNP weights are typically used in target-cohort PRS association analyses [81].

## Data Availability

No datasets were generated or analysed during the current study.
