# Supplementary material for: Evidence standards for multi-ancestry polygenic prediction
Source: Genome Biol. 2026 Jul 13;27:305. doi: 10.1186/s13059-026-04187-8 (PMC13644127; doi:10.1186/s13059-026-04187-8)
Supplement: Supplementary file 1 [file 13059_2026_4187_MOESM1_ESM.docx]

# Additional File 1.

# The logic behind the models and intuition of Bayesian multi-ancestry polygenic risk score methods

This supplementary text provides a method-by-method explanation of Bayesian software tools currently used for multi-ancestry polygenic risk score (PRS) construction. The goal is not to reproduce every implementation detail of each package, but to explain clearly what each method is trying to estimate, how it treats linkage disequilibrium (LD), how it borrows information across ancestries and how the final PRS is usually taken forward for association testing in a target cohort. The focus is on seven widely discussed methods: PRS-CSx, SDPRX, BridgePRS, JointPRS, MUSSEL, XPASS and X-Wing.

The equations in this document are mathematically faithful to the logic of the published models, but they are intentionally simpler than the full software-specific implementations.

## Contents Page

1. Common framework and notation……………………………………………………..………. 1
2. PRS-CSx ……………………………………………………..………………………………………………. 4
3. SDPRX……………………………………………………..………………………………………………….. 6
4. BridgePRS……………………………………………………..…………………………………………….. 8
5. JointPRS ……………………………………………………..………………………………………………. 10
6. MUSSEL……………………………………………………..……………………………………………….. 12
7. XPASS……………………………………………………..…………………………………………………… 14
8. X-Wing……………………………………………………..…………………………………………………. 16
9. Comparative interpretation across methods…………………………………………………18
10. How the final PRS is usually taken forward for association analysis……………….19
11. Practical summary…………………………………………………………………………………………20

## 1. Common framework and notation

### 1.1 What all of these methods are trying to do

All Bayesian PRS methods start from the same problem. A genome-wide association study (GWAS) usually reports, for each single nucleotide polymorphism (SNP), a *marginal* effect estimate: the association obtained when that SNP is tested one at a time. These marginal GWAS estimates are useful, but they are not the same thing as the underlying joint SNP effects needed for a polygenic score.

The reason is LD. Nearby SNPs are correlated, so the GWAS signal at one SNP partly reflects its own effect and partly reflects the effects of nearby SNPs that it tags. Bayesian PRS methods therefore try to move from an LD-blurred set of marginal signals towards a cleaner set of joint SNP weights.

A convenient generic representation is

$${\hat{\mathbf{b}}}^{\left( k \right)}\approx R^{\left( k \right)}\boldsymbol{\beta}^{\left( k \right)}+\boldsymbol{\varepsilon}^{\left( k \right)}.$$

Here:

- ${\hat{\mathbf{b}}}^{\left( k \right)}$ is the vector of observed marginal GWAS effect estimates in ancestry $k$ within one LD block.
- $R^{\left( k \right)}$ is the LD correlation matrix for that ancestry and block.
- $\boldsymbol{\beta}^{\left( k \right)}$ is the vector of underlying joint SNP effects that the model is trying to infer.
- $\boldsymbol{\varepsilon}^{\left( k \right)}$ is sampling noise.

The basic idea is therefore simple. The model proposes a set of underlying effects $\boldsymbol{\beta}^{\left( k \right)}$, passes them through the ancestry-specific LD matrix $R^{\left( k \right)}$ and asks whether the resulting pattern looks like the observed GWAS summary statistics. A prior is then added to express the belief that most SNP effects should be small, so that noisy effects are shrunk towards zero.

### 1.2 Marginal effects versus conditional effects

A marginal GWAS effect is the coefficient from a one-SNP-at-a-time regression. A conditional effect is the effect of one SNP after accounting for correlated SNPs nearby. In a region with LD, those two quantities can be very different.

For two SNPs, A and B, in ancestry $k$, the LD matrix is

$$R^{\left( k \right)}=\left( \begin{matrix} 1 & r^{\left( k \right)} \\ r^{\left( k \right)} & 1 \end{matrix} \right),$$

where $r^{\left( k \right)}$ is the correlation between the two SNPs in ancestry $k$. The diagonal entries are 1 because each SNP is perfectly correlated with itself. The off-diagonal entries are the ancestry-specific LD correlations.

Under this two-SNP model, the expected marginal GWAS effects are approximately

$$E\left[ \hat{b}_{A}^{\left( k \right)} \right]\approx\beta_{A}^{\left( k \right)}+r^{\left( k \right)}\beta_{B}^{\left( k \right)},$$

and

$$E\left[ \hat{b}_{B}^{\left( k \right)} \right]\approx r^{\left( k \right)}\beta_{A}^{\left( k \right)}+\beta_{B}^{\left( k \right)}.$$

These equations encode the key intuition. The marginal signal at SNP A contains A’s own effect plus the part of B’s effect that leaks into A through LD. The marginal signal at SNP B behaves analogously. Therefore a non-causal tag SNP can have a strong marginal association if it is highly correlated with a causal SNP.

### 1.3 Why ancestry-specific LD matters

Suppose SNP A is truly causal and SNP B is not, so that $\beta_{A}=0.10$ and $\beta_{B}=0$. If A and B are strongly correlated in ancestry 1, for example $r^{\left( 1 \right)}=0.90$, then the expected marginal signal at B is approximately

$$E\left[ \hat{b}_{B}^{\left( 1 \right)} \right]\approx0.90\times0.10=0.09.$$

If the same two SNPs are only weakly correlated in ancestry 2, for example $r^{\left( 2 \right)}=0.10$, then

$$E\left[ \hat{b}_{B}^{\left( 2 \right)} \right]\approx0.10\times0.10=0.01.$$

So the same underlying biology can produce a strong tag-SNP association in one ancestry and a weak tag-SNP association in another ancestry, purely because LD differs. This observation is central to every multi-ancestry method described below.

### 1.4 The generic Bayesian algorithm in words

Most summary statistics Bayesian PRS methods, regardless of the specific software package, can be described in the following steps.

First, the genome is divided into LD blocks, so that inference can be carried out one region at a time. Second, within each block the method uses ancestry-specific LD matrices to predict the pattern of marginal GWAS statistics that should arise from a candidate set of joint SNP effects. Third, it compares those predicted marginal effects to the observed GWAS effects. Fourth, it shrinks implausibly large or weakly supported effects towards zero using a prior that favours sparse or weak genetic architecture. Fifth, it repeats this update process until the posterior distribution is adequately explored or the optimisation has stabilised. The posterior means are then used as SNP weights.

The final PRS for individual $i$ is computed as

$$\text{PRS}_{i}=\sum_{j=1}^{m} G_{ij}w_{j},$$

where $G_{ij}$ is the genotype of individual $i$ at SNP $j$ and $w_{j}$ is the estimated SNP weight.

### 1.5 What changes in the multi-ancestry setting

Multi-ancestry methods keep the same LD-aware likelihood idea, but extend it in one of several ways.

1. They define one effect vector $\boldsymbol{\beta}^{\left( k \right)}$ per ancestry rather than a single universal effect vector.
2. They use one LD matrix $R^{\left( k \right)}$ per ancestry so that the forward model respects ancestry-specific tagging patterns.
3. They add some form of coupling across ancestries. This coupling may be a shared shrinkage prior, a multivariate prior with cross-population correlation, a transfer-learning step that uses one ancestry as an informed prior for another, or a combination stage that learns how much weight to assign to ancestry-specific PRSs.

The important conceptual point is that these methods do **not** usually assume that the observed marginal lead SNPs should be identical across populations. Instead, they assume that at least some of the *underlying* genetic architecture is related across populations, while allowing the *observed* marginal GWAS patterns to differ because LD, allele frequency and effect-size correlation differ across populations.

## 2. PRS-CSx

### 2.1 Core idea

PRS-CSx [20] is a multi-ancestry extension of PRS-CS [43]. Its central idea is to fit an ancestry-specific LD-aware likelihood for each population, while linking the per-SNP effects across populations through a shared “continuous-shrinkage” prior [20]. This makes PRS-CSx one of the clearest examples of the general Bayesian strategy, by separating the modelling of LD from the modelling of cross-population sharing.

### 2.2 How the model is set up

Within ancestry $k$, PRS-CSx uses the same generic likelihood described above:

$${\hat{\mathbf{b}}}^{\left( k \right)}\approx R^{\left( k \right)}\boldsymbol{\beta}^{\left( k \right)}+\boldsymbol{\varepsilon}^{\left( k \right)}.$$

The prior is what distinguishes PRS-CSx from single-ancestry methods. Schematically, for SNP $j$,

$$\beta_{j}^{\left( k \right)}\mid\psi_{j}\sim N\left( 0,\tau_{k}\psi_{j} \right).$$

Here:

- $\beta_{j}^{\left( k \right)}$ is the effect of SNP $j$ in ancestry $k$.
- $\psi_{j}$ is a SNP-specific local shrinkage parameter shared across ancestries.
- $\tau_{k}$ is an ancestry-specific global scale parameter.

The shared local parameter $\psi_{j}$ is the key device. If the data suggest that SNP $j$ is part of a genuine signal in one ancestry, then the model becomes more willing to preserve a non-zero effect at that SNP in other ancestries, even though the actual effect sizes can still differ by ancestry.

### 2.3 Intuition for the continuous-shrinkage prior

The continuous-shrinkage prior does not force SNPs into rigid categories such as exactly zero or definitely non-zero. Instead, it gives each SNP its own level of shrinkage. Most SNPs receive strong shrinkage and are pulled close to zero. A smaller number of SNPs are allowed to retain larger effects if the LD-aware likelihood strongly supports them.

This is appealing for highly polygenic traits, because it matches the empirical pattern seen in GWAS: many very small effects and a minority of larger effects.

### 2.4 How PRS-CSx handles differences in LD across ancestries

PRS-CSx does **not** share a single LD matrix across populations. Instead, it evaluates the likelihood separately in each ancestry using that ancestry’s own LD reference panel. This means that the same latent signal can produce different marginal patterns in different populations without creating a contradiction inside the model.

Consider the two-SNP example again. If SNP A is causal and SNP B is only a tag, B can look strongly associated in a European dataset and only weakly associated in an African dataset if B tags A much better in Europeans than in Africans. PRS-CSx treats that as expected behaviour under ancestry-specific LD, not as evidence that B itself is biologically important in one ancestry but not the other.

### 2.5 What the algorithm is doing in practice

PRS-CSx works block by block and uses Markov chain Monte Carlo updates, implemented through a Gibbs sampling scheme in the original framework [20]. In plain language, the sampler repeatedly asks, for each SNP in turn: given the current estimates for the neighbouring SNPs, how much signal remains that this SNP itself needs to explain? If little residual signal remains after accounting for LD with neighbouring SNPs, the prior shrinks the SNP strongly. If substantial residual signal remains, the SNP is allowed to keep a larger posterior effect.

This process is repeated across the genome and across ancestries. The final posterior means are exported as SNP weights.

### 2.6 A worked example

Suppose the available discovery GWAS are from European and East Asian samples and the target cohort is South Asian. PRS-CSx first fits European GWAS summary statistics using European LD and East Asian GWAS summary statistics using East Asian LD. It then uses the shared continuous-shrinkage prior to connect the SNP effects across the two discovery groups. The output is two posterior weight vectors, one anchored to the European evidence and one anchored to the East Asian evidence.

In practical analyses, these ancestry-specific posterior scores are often combined in a tuning dataset from the target population. If the South Asian target cohort is genetically closer, with respect to the architecture of the trait, to one discovery population than the other, the fitted combination tends to place more weight on the corresponding PRS. The standard weighted combination is

$$\text{PRS}_{\text{final}}=a_{\text{EUR}} \text{PRS}_{\text{EUR}}+a_{\text{EAS}} \text{PRS}_{\text{EAS}},$$

where the coefficients $a_{\text{EUR}}$ and $a_{\text{EAS}}$ are learned in a tuning set.

### 2.7 What is most distinctive about PRS-CSx

PRS-CSx is best understood as a method that borrows information **at the SNP level through shared shrinkage**, while letting each ancestry keep its own LD map and its own effect estimates. Its conceptual strength is simplicity: the likelihood explains ancestry-specific tagging patterns, whereas the prior encourages cross-population borrowing when the evidence is consistent.

## 3. SDPRX

### 3.1 Core idea

SDPRX is a two-population Bayesian method that is more explicit than PRS-CSx about the possible cross-population states of each SNP [22]. For each SNP, SDPRX asks whether the effect is null in both populations, specific to population 1, specific to population 2, or shared across both populations with correlation.

This makes SDPRX especially intuitive when the scientific question is not merely whether information should be borrowed across ancestries, but *what kind of cross-population sharing pattern is most plausible for each SNP*?

### 3.2 The likelihood

SDPRX uses an LD-aware multivariate normal likelihood that links the observed marginal GWAS effects in each population to the underlying SNP effects in each population. At the same intuitive level as the general model, this can be written as:

$${\hat{\mathbf{b}}}^{\left( 1 \right)}\approx R^{\left( 1 \right)}\boldsymbol{\beta}^{\left( 1 \right)}+\boldsymbol{\varepsilon}^{\left( 1 \right)}, {\hat{\mathbf{b}}}^{\left( 2 \right)}\approx R^{\left( 2 \right)}\boldsymbol{\beta}^{\left( 2 \right)}+\boldsymbol{\varepsilon}^{\left( 2 \right)}.$$

The important nuance is that SDPRX treats the pair of GWAS vectors jointly and allows their uncertainty structure to depend on both populations’ LD patterns. This is why the method can decide whether a discrepancy between populations is likely to be caused by different LD tagging or by a genuinely different effect pattern.

### 3.3 The prior: four SNP-level states

Schematically, for SNP $j$,

$$\left( \beta_{j}^{\left( 1 \right)},\beta_{j}^{\left( 2 \right)} \right)\sim\pi_{0} \delta_{\left( 0,0 \right)}+\pi_{1}F_{1}+\pi_{2}F_{2}+\pi_{12}F_{12}\left( \rho\right).$$

Here:

- $\delta_{\left( 0,0 \right)}$ means a point mass at zero in both populations.
- $F_{1}$ means non-zero only in population 1.
- $F_{2}$ means non-zero only in population 2.
- $F_{12}\left( \rho\right)$ means non-zero in both populations with correlation $\rho$.
- $\pi_{0}$, $\pi_{1}$, $\pi_{2}$ and $\pi_{12}$ are the probabilities of the four states.

This prior gives SDPRX a very direct biological interpretation. The model does not merely say that effects are partly shared; it assigns each SNP to a probabilistic cross-population category.

### 3.4 Why the off-diagonal shrinkage matters

A distinctive feature of SDPRX is that it shrinks the off-diagonal covariance in the likelihood to reduce inflation caused by mismatch between the GWAS sample and the LD reference panel [22] In plain language, the off-diagonal terms tell the model how strongly neighbouring SNPs are correlated. If the reference LD matrix slightly overstates those correlations, the model can become overconfident and spread signal too aggressively across high-LD SNPs. SDPRX therefore deliberately tones down those cross-SNP covariance terms. This is a conservative regularisation step. It does not remove LD from the model. Rather, it says that the exact off-diagonal LD values taken from the reference panel should not be trusted without limit.

### 3.5 LD blocks and Gibbs sampling

SDPRX partitions the genome into approximately independent LD blocks using the element-wise maximum of the two populations’ LD matrices. The reason behind this is straightforward; if two SNPs are strongly linked in either population, it is safer to keep them in the same block. Otherwise the assumption of block independence would be more likely to fail for one of the two ancestries.

After block construction, SDPRX uses a Markov chain Monte Carlo algorithm based on Gibbs sampling. The sampler repeatedly, updates the latent state of each SNP and its effect sizes conditional on the current values for all other SNPs. The output is the posterior mean effect in each population.

### 3.6 A worked example

Returning to the two-SNP example, with A causal and B non-causal. In population 1, A and B are highly correlated. In population 2, they are weakly correlated. Suppose B shows a strong marginal signal in population 1 but a near-zero marginal signal in population 2.

SDPRX can explain this pattern without declaring B population-specific. The ancestry-specific likelihood says that B’s population 1 signal is consistent with tagging A through strong LD, while the population 2 data show little direct evidence for B because LD is weak there. If this LD-based explanation fits well, the posterior favours either a shared effect on A or a null effect on B rather than a genuine population-specific B effect.

If, however, B remained strongly associated in population 2 despite weak LD with A, the shared-tagging explanation would no longer fit well and the model would move more probability towards a population-specific or jointly non-zero state for B.

### 3.7 What is most distinctive about SDPRX

SDPRX is most distinctive for two reasons. First, it makes the SNP-level cross-population states explicit. Second, it includes extra safeguards against LD-reference mismatch. It is therefore a good method to understand when the analysis is framed around the question: *is this locus shared, population-specific, or simply tagged differently because LD differs?*

## 4. BridgePRS

### 4.1 Core idea

BridgePRS takes a different philosophical approach from PRS-CSx and SDPRX. Instead of jointly modelling multiple ancestries in one symmetric Bayesian system, it uses a two-stage transfer strategy [21] It first learns a Bayesian ridge model in a better powered source population, then uses the resulting posterior as the prior for the target population.

This is why the name “BridgePRS” is appropriate: information is literally carried from one ancestry to another through a posterior-as-prior update.

### 4.2 Stage 1: fit the source ancestry

BridgePRS operates at the locus level. Within a locus, SNP effects are given a multivariate Gaussian prior. Schematically,

$$\boldsymbol{\beta}_{\mathcal{l}}^{\left( \text{source} \right)}\sim N\left( \mathbf{0},\Sigma_{\mathcal{l}} \right),$$

and the phenotype model at that locus is Gaussian as well. The choice of a Gaussian prior makes the update conjugate, which means that the posterior can be obtained analytically rather than through a heavy sampling algorithm.

In plain language, stage 1 asks: if the source ancestry GWAS and source LD structure are the main evidence available, what joint effect pattern across SNPs in this locus best predicts the phenotype while still applying ridge-like shrinkage to prevent overfitting?

### 4.3 Stage 2: update the target ancestry using the source posterior

After stage 1, BridgePRS carries forward not only point estimates of the source-ancestry effects, but also their uncertainty. The target ancestry is then modelled using that posterior as an informed prior. Schematically,

$$\boldsymbol{\beta}_{\mathcal{l}}^{\left( \text{target} \right)}\mid\text{source posterior}\sim N\left( \boldsymbol{\mu}_{\mathcal{l,}\text{source}},\Sigma_{\mathcal{l,}\text{source}}/\tau\right).$$

Here:

- $\boldsymbol{\mu}_{\mathcal{l,}\text{source}}$ is the stage-1 posterior mean in the source ancestry.
- $\Sigma_{\mathcal{l,}\text{source}}$ is the stage-1 posterior uncertainty.
- $\tau$ is a tuning parameter controlling how strongly the source ancestry influences the target ancestry.

Small values of $\tau$ make the target ancestry rely more on its own data. Large values of $\tau$ make the source ancestry prior more influential.

### 4.4 Why BridgePRS is less “fine-mapping-like”

BridgePRS is intentionally less aggressive about trying to isolate a tiny set of apparently causal variants. Instead, it keeps all variants in the locus and uses a multivariate Gaussian model to preserve tagging information [21]. This is a useful design choice when the causal variant is not directly observed or when the lead tag SNP changes across ancestries. In those situations, trying too hard to identify a single winner can harm prediction.

A good way to describe BridgePRS is therefore: it is a *predictive transfer* method rather than a *fine-mapping style* method.

### 4.5 What the algorithm is doing in practice

The software fits many locus-level models under different hyperparameter settings and different sets of included loci. It then forms genome-wide PRSs from these alternatives and uses ridge regression in a tuning dataset to decide how strongly each candidate score should contribute to the final PRS.

So, although the Bayesian core of BridgePRS is the two-stage posterior update, the final prediction layer also contains a supervised model-selection component.

### 4.6 A worked example

Imagine a source European GWAS with very large sample size and a smaller African target GWAS. In one locus, the causal variant is not directly genotyped, but several observed SNPs tag it reasonably well in Europeans and only imperfectly in Africans. A method that focuses too strongly on choosing one apparent lead SNP may transfer poorly, because the best tag differs between the two ancestries.

BridgePRS handles this by first learning the overall pattern of effects across the full European locus and then using that posterior pattern as prior information in the African stage. The African data are free to move away from the European solution if the African evidence demands it, but the model does not begin from scratch. It begins from a probabilistic summary of what the source population already taught it about the whole locus.

### 4.7 What is most distinctive about BridgePRS

BridgePRS is best thought of as a two-stage Bayesian ridge transfer method. Its signature feature is not an elaborate SNP-level shared prior across all ancestries, but a sequential update: Having first learned a genome-wide, LD-aware ridge posterior for SNP effects in the source ancestry, this estimate is then updated in the target ancestry while preserving locus-wide structure.

## 5. JointPRS

### 5.1 Core idea

JointPRS was designed to handle two problems that frequently arise in non-European prediction: Limited non-European GWAS sample size and limited non-European tuning data [81]. It combines a continuous-shrinkage prior with an internally estimated cross-population genetic correlation structure and a data-adaptive strategy for deciding how much to rely on tuning data when such data are available [81].

The method therefore sits conceptually between PRS-CSx and more explicitly correlation-driven models. It keeps the continuous-shrinkage machinery of PRS-CSx, but adds an explicit genetic-correlation layer.

### 5.2 The shared structure across populations

A schematic way to write the JointPRS idea is

$$\text{Cov}\left( \beta_{j}^{\left( k_{1} \right)},\beta_{j}^{\left( k_{2} \right)} \right)\propto\rho_{k_{1}k_{2}} \Psi_{j}.$$

Here:

- $\rho_{k_{1}k_{2}}$ is the estimated genetic correlation between populations $k_{1}$ and $k_{2}$.
- $\Psi_{j}$ is a shared SNP-level scale parameter that is itself given a continuous-shrinkage prior.

This says that the extent to which populations share information at SNP $j$ depends on two things: the overall tendency of those populations to share genetic effects for the trait and the extent to which SNP $j$ is allowed by the shrinkage prior to carry non-trivial signal.

### 5.3 Why the estimated genetic correlation matters

PRS-CSx couples ancestries through shared shrinkage, but it does not explicitly estimate a cross-population genetic correlation structure as part of the model. JointPRS does. This distinction matters most when tuning data are absent or very limited.

Intuitively, if two populations have high genetic correlation for the trait, then evidence from one population is more informative for the other. If the correlation is lower, borrowing should be weaker. JointPRS tries to learn this relation rather than leaving it implicit.

The published implementation emphasises automatically estimated chromosome-wise cross-population genetic correlations, rather than relying entirely on external estimates [81]. The approach is supposed to improve robustness.

### 5.4 The two data scenarios: no tuning data versus tuning data available

JointPRS has two main operating modes.

When no tuning data are available, JointPRS-auto builds the PRS using GWAS summary statistics and LD reference panels only. In this setting the internal genetic-correlation structure is particularly important because it governs how strongly information can be shared across populations.

When tuning data are available, JointPRS uses a data-adaptive strategy. One part of the procedure behaves like a meta-analysis style combination and another part uses a more conventional tuning-based combination. The method then chooses the more appropriate solution for the available data. The rationale for this is practical: a small tuning dataset can be noisy, so one should not automatically trust a fully tuned solution more than a stable meta-like solution.

### 5.5 What the algorithm is doing in practice

JointPRS uses a Bayesian sampling-based fitting procedure related to the continuous-shrinkage family. Within LD blocks, the algorithm repeatedly updates SNP effects while also updating the parameters that control the cross-population sharing structure. The published implementation uses an MCMC scheme, with the aim of approximating the joint posterior over effect sizes and shrinkage-related parameters [81].

In intuitive terms, JointPRS repeatedly asks two coupled questions. First, what effect size at this SNP is needed to explain the ancestry-specific GWAS data once LD has been accounted for? Second, how much should the answer be pulled towards the corresponding effect in another population, given the estimated genetic correlation between the populations?

### 5.6 A worked example

Consider European, African, South Asian and Admixed American summary statistics for a lipid trait, with only a very small South Asian tuning set. PRS-CSx can still share information across populations, but it has no explicit mechanism for learning how strongly the trait’s genetic architecture is correlated between the populations. JointPRS explicitly estimates those cross-population correlations and uses them to decide how much sharing is warranted.

If the South Asian tuning set is tiny and noisy, a purely tuning-driven linear combination may become unstable. JointPRS addresses this by combining a meta-like route and a tuning-driven route and by adaptively choosing the one that appears more reliable for the available data.

### 5.7 What is most distinctive about JointPRS

JointPRS is most distinctive for two reasons. First, it incorporates an explicit genetic-correlation structure into a continuous-shrinkage framework. Second, it was built to remain usable when the tuning data situation is poor, which is a common reality in under-represented populations.

## 6. MUSSEL

### 6.1 Core idea

MUSSEL combines two ideas: a Bayesian hierarchical model for joint ancestry-specific SNP effects and an ensemble-learning step for combining multiple PRSs [24]. The name reflects those two parts: MUSS for the multivariate spike-and-slab Bayesian model and EL for the ensemble learner.

The guiding intuition is that no single tuning-parameter setting or ancestry source is guaranteed to be best across all traits and populations. MUSSEL therefore creates several plausible PRSs and then combines them using a supervised ensemble.

### 6.2 The multivariate spike-and-slab model

For SNP $j$, MUSSEL treats the vector of effects across ancestries,

$$\boldsymbol{\beta}_{j}=\left( \beta_{j}^{\left( 1 \right)},\ldots,\beta_{j}^{\left( K \right)} \right),$$

as arising from a multivariate spike-and-slab prior. Schematically,

$$\boldsymbol{\beta}_{j}\sim\pi_{0} \delta_{\mathbf{0}}+\left( 1-\pi_{0} \right)N\left( \mathbf{0},\Sigma_{\beta} \right).$$

Here:

- the *spike* means the SNP is near zero or exactly zero across ancestries,
- the *slab* means the SNP has non-trivial effects,
- and the multivariate covariance $\Sigma_{\beta}$ governs how those non-zero effects co-vary across ancestries.

This prior is conceptually different from PRS-CSx. PRS-CSx uses a continuous shrinkage distribution for all SNPs, whereas MUSSEL more explicitly separates likely-null SNPs from likely non-null SNPs.

### 6.3 The ancestry-specific likelihood

MUSSEL still uses ancestry-specific LD in the likelihood. Thus, as in the other methods, a SNP can look important in one ancestry and less important in another simply because the LD pattern differs. The spike-and-slab prior does not replace the LD-aware likelihood; it sits on top of it.

This point is important. MUSSEL is not saying “if the marginal GWAS signal differs, then the biology must differ.” Rather, it says “first explain marginal differences through ancestry-specific LD; then decide whether the remaining evidence suggests a truly shared or non-shared effect pattern.”

### 6.4 The ensemble-learning step

After the Bayesian step has generated a family of PRSs under different parameter settings and across different ancestry groups, MUSSEL combines them through a super learner. Schematically,

$$\text{PRS}_{\text{final}}=\sum_{t=1}^{T} a_{t} \text{PRS}^{\left( t \right)},$$

where the weights $a_{t}$ are learned from validation data.

This ensemble layer is a central part of the method. The Bayesian step produces several potentially useful candidate scores and the super learner is tasked with determining how much each should contribute.

### 6.5 What the algorithm is doing in practice

MUSSEL first fits the Bayesian hierarchical model to summary statistics and LD reference panels across ancestries. It does this under a range of tuning parameter settings, generating multiple ancestry-specific candidate PRSs. It then computes these scores in a validation dataset and trains the super learner to obtain a weighted combination with improved prediction.

A useful way to think about MUSSEL is that it hedges against model uncertainty twice: first through Bayesian modelling of SNP effects and second through supervised ensembling of multiple PRSs.

### 6.6 A worked example

Suppose a trait is highly polygenic and the available discovery GWAS include European, African, East Asian and Hispanic/Latino samples. One set of tuning parameters may work well when the effect-size distribution is relatively dense, whereas another may work better when the trait is effectively sparser in a particular ancestry because of GWAS sample-size limitations. Instead of forcing the analyst to choose one tuning configuration in advance, MUSSEL keeps multiple candidate solutions and lets the ensemble learner decide how to combine them.

This is especially attractive when there is no reason to believe that one Bayesian parameterisation will perform best across all ancestries and all traits.

### 6.7 What is most distinctive about MUSSEL

MUSSEL is the clearest example of a multi-ancestry Bayesian method that does not stop at posterior SNP weights. It explicitly adds a second layer of learning over candidate PRSs. Conceptually, it says that although *joint SNP modelling is useful, the final predictor may still be improved by supervised aggregation of multiple Bayesian scores.*

## 7. XPASS

### 7.1 Core idea

XPASS is the summary statistics member of a broader cross-population framework that also includes an individual-level method called XPA [23]. XPASS uses trans-ancestry genetic correlation to borrow information from an auxiliary population into a target population while accounting for the fact that LD differs between them.

The method is older than most of the other tools reviewed here, but it introduced an important design principle that remains influential: *borrowing should depend on estimated cross-population genetic correlation and the target population should not be forced to copy the auxiliary population when that correlation is weak.*

### 7.2 The modelling intuition

The starting point of XPASS is similar to LDpred-inf, but extended to two populations. It estimates heritability in each population and co-heritability between populations, then uses those quantities to derive a posterior mean effect for the target population.

At an intuitive level,

$$E\left( \boldsymbol{\beta}_{\text{target}}\mid\text{data} \right)\approx\text{LD-corrected target evidence}+\rho\times\text{LD-corrected auxiliary evidence},$$

where $\rho$ is the trans-ancestry genetic correlation.

This equation should be read conceptually rather than literally. It says that the posterior mean for the target population is a compromise between what the target GWAS says on its own and what the auxiliary GWAS says, scaled by how genetically correlated the two populations are for the trait.

### 7.3 How LD enters XPASS

XPASS uses ancestry-specific LD information for the target and auxiliary populations and uses those LD matrices when building the posterior mean. In the implementation described by [23], the genome is approximated by nearly independent LD blocks for PRS construction, while heritability and co-heritability estimation can use broader chromosome-wide LD information. The software documentation also notes that chromosome-wide LD is used for estimating heritability and co-heritability, which can reduce standard errors but requires careful adjustment for population structure in the reference panel.

The practical intuition is straightforward: If the auxiliary population is extremely well powered, it contains useful information, but that information must be translated through the correct LD map rather than copied directly.

### 7.4 XPASS+

XPASS+ extends the framework by allowing large population-specific effects to be added on top of the shared polygenic background. This makes the method more flexible when most of the architecture is shared but a subset of variants has notably larger or more population-specific effects.

Conceptually, the model separates two components:

1. a shared polygenic component that can be borrowed across populations using the estimated genetic correlation and
2. a population-specific large-effect component that is not forced to obey the same sharing pattern.

### 7.5 What the algorithm is doing in practice

In practice, XPASS estimates the cross-population variance components, builds posterior mean effects for the target population from summary statistics and LD reference information and then computes the PRS in the target sample. Compared with later continuous-shrinkage or mixture-based methods, the logic is closer to an analytically derived best linear predictor than to a heavy sampling model.

This gives XPASS a different flavour from PRS-CSx, SDPRX and JointPRS. It is more variance-component driven and less centred on per-SNP adaptive shrinkage distributions.

### 7.6 A worked example

Suppose a target Chinese GWAS is modest in size and a European GWAS is much larger. If the estimated trans-ancestry genetic correlation is high, XPASS will allow the European summary statistics to contribute substantially to the posterior mean for the Chinese effects. If the estimated genetic correlation is close to zero, the European contribution is downweighted and the method behaves more like a target-only predictor.

Thus, unlike a naive meta-analysis, XPASS has a built-in brake, in that it borrows auxiliary information only to the extent that the variance-component model suggests the underlying genetic architecture is actually shared.

### 7.7 What is most distinctive about XPASS

XPASS is most distinctive as a cross-population summary statistics framework built around genetic correlation and variance components. It is less flexible than some newer methods at representing highly heterogeneous local sharing patterns, but it remains conceptually important because it formalised the idea that cross-population borrowing should be controlled by estimated genetic correlation rather than by sample size alone.

## 8. X-Wing

### 8.1 Core idea

X-Wing starts from the observation that cross-population sharing may be highly local rather than uniform across the genome [25]. Some regions may carry portable effects across populations, whereas others may be much more population-specific. X-Wing therefore proceeds in three steps:

1. identify regions showing positive local genetic correlation between populations,
2. use those regions as annotations in a Bayesian shrinkage model and
3. combine population-specific PRSs using GWAS summary statistics alone.

This makes X-Wing a region-aware method rather than a purely global-sharing method.

### 8.2 Step 1: detect locally portable regions

X-Wing extends the LOGODetect framework to scan the genome for regions showing local genetic correlation between populations. The important conceptual shift is from asking “how genetically correlated are these populations overall?” to asking “which parts of the genome show shared signal?”

A region that shows positive local genetic correlation is treated as more likely to contain portable effects. A region showing weak or absent local correlation is treated more cautiously.

### 8.3 Step 2: annotation-dependent shrinkage

Once locally portable regions have been identified, X-Wing feeds that information into a Bayesian shrinkage model. Schematically,

$$\beta_{jk}\sim N\left( 0,\phi\psi_{j} \lambda_{f\left( j \right),k} \right).$$

Here:

- $\phi$ is a global shrinkage parameter,
- $\psi_{j}$ is a SNP-specific local shrinkage parameter,
- $\lambda_{f\left( j \right),k}$ is an annotation-dependent parameter that depends on whether SNP $j$ lies in a region annotated as locally portable for population $k$.

The intuition is extremely clear. SNPs in regions believed to be portable receive less shrinkage. SNPs in regions with little evidence of portability receive more shrinkage. Thus X-Wing does not treat the whole genome as equally transferable across ancestries.

### 8.4 Step 3: combine scores without individual-level tuning data

The third distinctive component of X-Wing is its summary statistics based repeated learning strategy for combining multiple PRSs. Schematically,

$$\text{PRS}_{\text{final}}=\sum_{k=1}^{K} a_{k} \text{PRS}^{\left( k \right)},$$

but unlike standard tuning-set regression, the coefficients $a_{k}$ are estimated from summary statistics alone rather than from a held-out individual-level validation sample.

This is a major practical advantage when a clean target-ancestry tuning dataset is not available.

### 8.5 What the algorithm is doing in practice

X-Wing first scans for local cross-population correlation signals, then trains a Bayesian PRS model that uses these local annotations to modulate shrinkage and finally estimates score-combination weights through repeated learning with summary statistics. In software terms, the framework is often described through the components LOGODetect, PANTHER and LEOPARD, but the key scientific logic is exactly the three-step process above.

### 8.6 A worked example

Suppose a trait has one locus where European and East Asian studies both point to the same underlying biology, even though the top tag SNP differs because the LD patterns differ. X-Wing is designed to identify that region as locally portable. Once that happens, the SNPs in that region receive less shrinkage in the subsequent Bayesian PRS model. The final PRS therefore preserves more of the shared signal in that locus than a method that assigns the same shrinkage profile everywhere in the genome.

Conversely, if another region shows weak or inconsistent local sharing, X-Wing increases shrinkage there so that population-specific noise is less likely to contaminate the transferred PRS.

### 8.7 What is most distinctive about X-Wing

X-Wing is most distinctive because it moves the question of ancestry transferability from a global scale to a local scale. It effectively says: *do not assume the whole genome is equally portable; find the portable regions first, then let them influence shrinkage and score combination.*

## 9. Comparative interpretation across methods

### 9.1 The main axis of difference: where cross-population sharing is imposed

The methods above differ less in their basic LD-aware likelihood than in the stage at which they impose or learn cross-population sharing.

PRS-CSx imposes sharing through a shared continuous-shrinkage prior at the SNP level. SDPRX imposes sharing through a mixture prior over explicit two-population SNP states. BridgePRS imposes sharing sequentially, by using the source posterior as the target prior. JointPRS imposes sharing through a continuous-shrinkage framework augmented by an explicit genetic-correlation structure. MUSSEL imposes sharing through a multivariate spike-and-slab model and then adds a supervised ensemble across candidate PRSs. XPASS imposes sharing through variance components and trans-ancestry genetic correlation. X-Wing imposes sharing in a region-specific manner, using local genetic correlation annotations.

### 9.2 The second axis of difference: how much tuning data is needed

A second important distinction is whether the method relies heavily on individual-level tuning data. PRS-CSx is often followed by a tuning-based combination of ancestry-specific PRSs. BridgePRS and MUSSEL explicitly contain tuning-dependent steps. JointPRS was built to remain useful even when tuning data are absent or very limited. X-Wing is especially notable because it can estimate combination weights from summary statistics alone. XPASS also works naturally in a summary statistics-only setting. SDPRX can be used without a tuning set, although the original paper also discusses an optional validation-based convex combination of its output weights.

### 9.3 The third axis of difference: global versus local sharing

Some methods mostly learn a *global* degree of sharing across ancestries. XPASS and JointPRS are the clearest examples. Other methods allow more local or SNP-specific heterogeneity. SDPRX does this by applying explicit SNP-level state assignments, PRS-CSx does it through SNP-level shrinkage, X-Wing does it through local genetic correlation annotations. BridgePRS takes a locus-level transfer perspective and MUSSEL allows heterogeneity to be expressed both in the Bayesian layer and in the ensemble layer.

### 9.4 What all of the methods have in common

Despite these differences, all of the methods share one central principle: they model ancestry-specific LD rather than pretending that marginal GWAS hits are directly comparable across populations. This is the fundamental reason these methods can perform better than simple approaches that combine summary statistics without carefully accounting for LD.

## 10. How the final PRS is usually taken forward for association analysis

The software packages above estimate SNP weights. The actual statistical test of PRS-trait association is usually a separate regression in the target cohort.

For a quantitative phenotype, the common model is

$$Y_{i}=\alpha+\gamma\text{PRS}_{i}+\mathbf{c}_{i}^{T}\boldsymbol{\theta}+\epsilon_{i},$$

where:

- $Y_{i}$ is the phenotype for individual $i$,
- $\text{PRS}_{i}$ is the polygenic score,
- $\mathbf{c}_{i}$ is a vector of covariates such as age, sex, ancestry principal components, recruitment centre and genotyping batch,
- $\gamma$ is the PRS association parameter of interest.

For a binary phenotype, the corresponding logistic regression is

$$\log\left( \frac{P\left( Y_{i}=1 \right)}{1-P\left( Y_{i}=1 \right)} \right)=\alpha+\gamma\text{PRS}_{i}+\mathbf{c}_{i}^{T}\boldsymbol{\theta}.$$

The PRS is often standardised within the analysis sample so that $\gamma$ can be interpreted as the change in phenotype per one standard deviation increase in PRS. In related samples or in datasets with strong structure, mixed models may be preferable. The key point is that the multi-ancestry Bayesian method typically ends when the SNP weights have been estimated. The association analysis that follows is generally the same regression framework used for any other PRS.

## 11. Practical summary

The seven methods reviewed here solve the same broad problem but embody different views of what it means for genetic effects to be shared across populations. PRS-CSx assumes that sharing can be encouraged through a shared continuous-shrinkage prior. SDPRX assumes that SNPs fall into a small set of interpretable cross-population states and explicitly models those states. BridgePRS assumes that a strong source population can provide a useful posterior prior for a weaker target population. JointPRS assumes that explicit cross-population genetic correlation should be built into the Bayesian model and that tuning data may be too sparse to trust uncritically. MUSSEL assumes that joint Bayesian modelling should be followed by ensemble learning because no single score is uniformly best. XPASS assumes that trans-ancestry genetic correlation and variance components provide the right foundation for cross-population borrowing. X-Wing assumes that transferability is local rather than uniform and should be learned region by region. Each corresponds to a different scientific assumption about (i) how much of genetic architecture is shared across ancestries, (ii) how that sharing varies across the genome and (iii) how much one should rely on external tuning data. Understanding those assumptions is essential for selecting an appropriate method and for interpreting the resulting PRS in a target population.
